# Improved allele-specific single-cell copy number estimation in low-coverage DNA-sequencing

**DOI:** 10.1093/bioinformatics/btae506

**Published:** 2024-08-12

**Authors:** Samson Weiner, Bingjun Li, Sheida Nabavi

**Affiliations:** School of Computing, University of Connecticut, Storrs, CT 06082, United States; School of Computing, University of Connecticut, Storrs, CT 06082, United States; School of Computing, University of Connecticut, Storrs, CT 06082, United States; Institute for Systems Genomics, University of Connecticut, Storrs, CT 06082, United States

## Abstract

**Motivation:**

Advances in whole-genome single-cell DNA sequencing (scDNA-seq) have led to the development of numerous methods for detecting copy number aberrations (CNAs), a key driver of genetic heterogeneity in cancer. While most of these methods are limited to the inference of total copy number, some recent approaches now infer allele-specific CNAs using innovative techniques for estimating allele-frequencies in low coverage scDNA-seq data. However, these existing allele-specific methods are limited in their segmentation strategies, a crucial step in the CNA detection pipeline.

**Results:**

We present SEACON (Single-cell Estimation of Allele-specific COpy Numbers), an allele-specific copy number profiler for scDNA-seq data. SEACON uses a Gaussian Mixture Model to identify latent copy number states and breakpoints between contiguous segments across cells, filters the segments for high-quality breakpoints using an ensemble technique, and adopts several strategies for tolerating noisy read-depth and allele frequency measurements. Using a wide array of both real and simulated datasets, we show that SEACON derives accurate copy numbers and surpasses existing approaches under numerous experimental conditions, and identify its strengths and weaknesses.

**Availability and implementation:**

SEACON is implemented in Python and is freely available open-source from https://github.com/NabaviLab/SEACON and https://doi.org/10.5281/zenodo.12727008.

## 1 Introduction

Extensive genetic diversity exists within tumor cell populations through the accumulation of somatic alterations (Burrell *et al.* 2013). Copy number aberrations (CNAs) are highly prevalent in cancer and play a key role in shaping the heterogeneous genomic landscape within tumors (Zack *et al.* 2013). Intra-tumor heterogeneity (ITH) remains a critical challenge for clinical decision-making in precision medicine (McGranahan and Swanton 2015) and necessitates the genome-wide profiling of CNAs. Advances in next-generation sequencing have made possible the characterization of CNAs at increasing resolution.

While numerous computational methods have been developed for CNA detection from bulk DNA-sequencing (Gabrielaite *et al.* 2021), the task of deconvoluting aggregated copy number signals across multiple subclones remains challenging (Xiao *et al.* 2020). Single-cell DNA sequencing (scDNA-seq) has emerged as a powerful means of CNA profiling at the single-cell level, thereby circumventing the deconvolution problem. However, technical errors and shallow sequencing coverage pose new challenges for CNA detection (Navin 2014).

For well over a decade, CNA detection from scDNA-seq has been an active problem with over a dozen proposed methods. These methods typically follow a four-step pipeline (Mallory *et al.* 2020) summarized as follows: (i) a reference genome is divided into bins and the number of aligned reads is counted within each bin; (ii) biases in the read counts, e.g. from GC-content and mappability, are corrected; (iii) breakpoints between two adjacent genomic regions with differing copy number are identified, effectively partitioning the genome into discrete segments; (iv) segments are assigned an absolute integer copy number. The earliest methods applied to single cells were originally designed for other data modalities (Shah *et al.* 2006), but more recent approaches are customized for the specific aspects of scDNA-seq data. In particular, a great deal of attention has been given to the challenging task of segmentation. Most methods make use of tried-and-true signal processing techniques, including Circular Binary Segmentation (CBS) (Garvin *et al.* 2015, Wang *et al.* 2018, 2020) and Hidden Markov Models (HMM) (Shah *et al.* 2006, Bakker *et al.* 2016, Hui and Nielsen 2022). To overcome noisy read count signals, some recent methods have taken a global segmentation approach that leverages breakpoint signals shared across cells (Wang *et al.* 2020, Feng *et al.* 2021, Ruohan *et al.* 2022, Liu *et al.* 2024) as cells from the same subclone are expected to share many breakpoints. However, CNAs occurring in low frequency may be overlooked.

Nearly all existing single-cell CNA detection methods are limited to the inference of total copy number, defined as the sum of the allele-specific copy numbers belonging to each homologous chromosome. Recent large-scale cancer studies have indicated that certain types of genomic aberrations are undetectable from total copy number profiles yet play a significant role in cancer development (Van Loo *et al.* 2010, Zack *et al.* 2013). This includes copy-neutral loss of heterozygosity (LOH) events, the detection of which has been used as a prognostic factor across multiple cancer types (Zhang *et al.* 2012). The challenge in detecting allele-specific CNAs from low coverage scDNA-seq is that standard approaches for calculating the B-allele frequency (BAF) perform poorly. Recently, the CHISEL algorithm (Zaccaria and Raphael 2021) was the first method developed for allele-specific profiling from scDNA-seq, followed shortly thereafter by Alleloscope (Wu *et al.* 2021) and SIGNALS (Funnell *et al.* 2022). All three methods overcome the challenge of computing BAFs in low coverage by pooling information across single-nucleotide polymorphisms (SNPs) positions and cells to estimate the phase of short haplotype blocks. While this approach presents new opportunities for single-cell CNA analysis, there are limitations in the segmentation procedure used by these methods. Alleloscope segments a matched-bulk sample rather than the single cells, and is thus far less sensitive to low frequency CNAs in the population, while SIGNALS uses segments produced by HMMCopy (Shah *et al.* 2006) thereby ignoring shared breakpoints. CHISEL identifies bins with identical copy number state globally across cells, but ignores the continuity of segments. Additionally, it is ill-equipped to handle noisy BAF measurements, limiting effective use to datasets with sufficient total coverage or huge bin sizes, sacrificing resolution.

**Figure 1.**
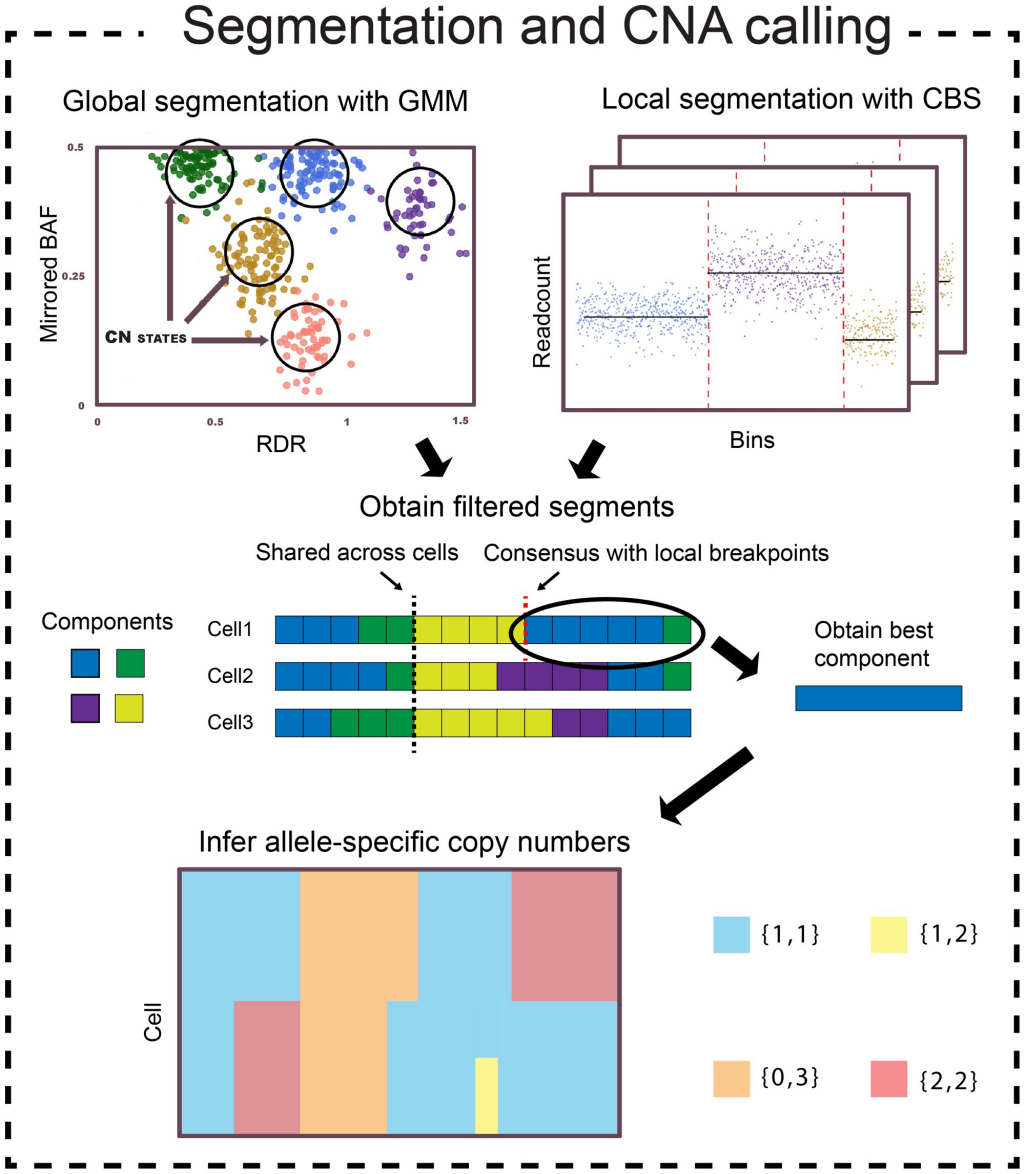
The segmentation workflow of SEACON. SEACON uses a Gaussian Mixture Model to identify latent copy number states and breakpoints, which are filtered using locally derived breakpoints. The allele-specific copy numbers are estimated by minimizing the distance between inferred segments and latent copy number states.

In this paper, we introduce SEACON (Single-cell Estimation of Allele-specific COpy Numbers), a framework for single-cell allele-specific copy number estimation (Fig. 1). SEACON uses the same BAF estimation approach of CHISEL but introduces a novel segmentation heuristic combining global and local breakpoint detection with other features designed to reduce noise and produce higher quality segments. First, a Gaussian Mixture Model (GMM) is used to identify the latent allele-specific copy number states and a set of candidate breakpoints for each cell. To overcome noisy measurements, we implement a post hoc weighted merging of the GMM components. Next, SEACON uses an ensemble segmentation approach in which the candidate breakpoints are first filtered according to their global frequency across cells, and then refined using the CBS algorithm locally for each cell. Given the refined segments, SEACON minimizes the distance between segment means and allele-specific copy number states. Using a comprehensive simulation pipeline, we show that SEACON matches or outperforms existing state-of-the-art methods in inferring copy number states, estimating ploidy, and detecting breakpoints in many experimental conditions. Our simulation study also represents the first end-to-end benchmarking of single-cell allele-specific CNA calling using synthetic DNA sequencing reads.

On three real breast cancer datasets, we demonstrate instances where (i) SEACON produces more reliable results than both CHISEL and state-of-the-art total copy number methods using weak BAF signals, (ii) SEACON and CHISEL generate unreliable results if no reliable BAF signals can be derived, and (iii) SEACON and CHISEL produce equivalent results under ideal conditions.

## 2 Materials and methods

Suppose we are given scDNA-seq data for *n* cells and a reference genome that is divided into *m* fix-sized bins. The objective of SEACON is to estimate allele-specific copy numbers $\left\{c_{ij}^{\prime },c_{ij}^{\prime \prime }\right\}$ for each cell *i* and bin *j*, where 1≤i≤n and 1≤j≤m. This is achieved using two measurements which are derived from the sequencing data: read counts and BAFs. Read count, denoted *X_ij_*, measures the volume of reads aligned to *j* and is proportional to the total copy number $c_{ij}^{\prime }+c_{ij}^{\prime \prime }$, while BAF, denoted *Y_ij_*, is an estimate of allelic imbalance equal to $\frac{c_{ij}^{\prime }}{c_{ij}^{\prime }+c_{ij}^{\prime \prime }}$ or $\frac{c_{ij}^{\prime \prime }}{c_{ij}^{\prime }+c_{ij}^{\prime \prime }}$. We also derive the alternative forms of these measurements: the read count ratio (RDR) ${\hat{X}}_{ij}=\frac{X_{ij}}{{\bar{X}}_{i}}$, where ${\bar{X}}_{i}$ is the mean read count of cell *i*, normalizes the read count of each bin to the cell coverage baseline, and the mirrored BAF (mBAF) ${\hat{Y}}_{ij}=\min\{Y_{ij},1-Y_{ij}\}$ ensures consistent values across bins with the same allele-specific copy number state (e.g. 2,3 versus 3,2) (Staaf *et al.* 2008).

### 2.1 Real data preprocessing

A number of preprocessing and quality control steps are required to obtain read counts and BAFs from the raw data (Supplementary Fig. S1). First, SEACON computes a raw cell-by-bin read count matrix, denoted $R_{n\times m}$. Following standard practices (Ruohan *et al.* 2022), the reads are first aligned to the reference genome using BWA (Li and Durbin 2010), then filtered for quality and sorted using Samtools (Danecek *et al.* 2021). After diving the reference genome into *m* consecutive bins, SEACON masks bins with extreme GC content (<0.2 or >0.8) or poor mappability (<0.9) from downstream analysis. This process removes error-prone regions while retaining the majority of known cancer-related genes (see Supplementary Section S2.1).

Next, SEACON integrates the BAF estimation procedure of CHISEL to obtain a BAF matrix, denoted $Y_{n\times m}$. This requires variant allele information in the form of phased heterozygous SNPs of the sampled individual. Using either a pseudo-bulk sample built from normal cells in the dataset or a matched-normal sample, we identify a set of SNPs using bcftools (Danecek *et al.* 2021), filter the set for heterozygous sites, and then phase them using Eagle2 (Loh *et al.* 2016).

### 2.2 Read count normalization

SEACON identifies healthy diploid cells within the sample to serve as coverage baselines for normalizing the raw read counts, implicitly accounting for mappability biases, GC content biases, and other latent factors. Normal cells are identified using the gini coefficient over the read counts (Wang *et al.* 2020), a measure of inequality among the values of a frequency distribution (Supplementary Note S1.1).

Let D⊂{1,…,n} be the set of indexes for normal cells. For each bin *j*, we compute a normalization factor *λ_j_* representing the expected deviation in read count at that bin as

(1) $$\lambda_{j}=\frac{\sum_{d\in D}R_{dj}/{\bar{R}}_{d}}{\left|D\right|},$$

where ${\bar{R}}_{d}$ is the mean read count for normal cell *d*. Given the set of normalization factors, the normalized read count for cell *i* and bin *j* is computed as $X_{ij}=\frac{R_{i,j}}{\lambda_{j}}$. Note that in the absence of normal cells, each *λ_j_* is computed using the average GC content and mappability of bin *j* (Dong *et al.* 2020).

### 2.3 Combined global and local segmentation

#### 2.3.1 Global segmentation with GMM

Consider the entire set of *n *×* m* genomic bins as existing within a 2D feature space (the read counts and BAFs) and that these features are distributed according to a finite set of latent allele-specific copy number states. To both detect breakpoints between continuous segments with shared copy number states and to infer integer allele-specific copy numbers, we use a Gaussian Mixture Model (GMM) across the feature space to cluster bins into distinct copy number states. In particular, we assume all bins across the entire genome of any cell which have the same latent copy number state follow the same Gaussian distribution. This follows from two observations: (i) the measurements observed over a genomic region is directly correlated with the underlying copy number of that region, and (ii) measurements from cells sequenced under the same experimental conditions are expected to follow similar variance.

Suppose bin *j* from cell *i* has copy number state k={c′,c″}, and let $V_{ij}={\left({\hat{X}}_{ij},{\hat{Y}}_{ij}\right)}^{⊤}$. We assume

(2) $$V_{ij}∼N(\mu_{k}={\left({\hat{X}}_{k},{\hat{Y}}_{k}\right)}^{⊤},\Sigma_{k}),$$

where ${\hat{X}}_{k}$ and ${\hat{Y}}_{k}$ are the mean RDR and mBAF of bins with copy number state *k*, respectively, and Σ_*k*_ is the variance. If *k* is known, then the expected mBAF is ${\hat{Y}}_{k}=\frac{\min c\prime ,c\prime \prime }{c\prime +c\prime \prime }$. Because the read count is proportional to the total copy number, the expected RDR is given by ${\hat{X}}_{i}=p_{i}(c\prime +c\prime \prime )$ where *p_i_* is the cell ploidy.

Suppose there are *K* distinct copy number states. The probability density over *V_ij_* is given by

(3) $$P(V_{ij}|\Theta )=\sum_{k=1}^{K}\psi_{k}f(V_{ij}|\Theta ,q_{ij}=k)=\sum_{k=1}^{K}\psi_{k}f(V_{ij}|\mu_{k},\Sigma_{k}),$$

(4) $$f(V_{ij}|\mu_{k},\Sigma_{k})=\frac{1}{2\pi \sqrt{\Sigma_{k}}}\text{exp}(-\frac{1}{2}{\left(V_{ij}-\mu_{k}\right)}^{⊤}\Sigma_{k}^{-1}(V_{ij}-\mu_{k})),$$

where *ψ_k_* is the weight of the *k*th Gaussian, $\Theta =\{\psi_{1},\ldots ,\psi_{k},\mu_{1},\ldots ,\mu_{k},\Sigma_{1},\ldots ,\Sigma_{k}\}$ are the model parameters, and *q_ij_* is the copy number state of cell *i* and bin *j*. We wish to find values of Θ that maximize the likelihood of the dataset by solving the following optimization problem:

(5) $$\underset{\Theta }{\max}P(X,Y|\Theta )=\underset{\Theta }{\max}\prod_{i,j}P(V_{i,j}|\Theta ).$$

We solve this problem using a standard EM algorithm.

SEACON selects an initial estimate on the number of components, or distinct copy number states, to be that which minimizes a standard Bayesian Information Criterion (BIC). However, several aspects of the feature space, including high variance in read count and BAF measurements and an extremely uneven distribution of copy number states, may result in over-clustering. To correct for this, we adopt a post hoc weighted component merging based on a simple distance threshold. For additional details, see Supplementary Note S1.2.

For every cell *i*, we compute a set of candidate breakpoints from the component assignments as $\left\{j|1\leq j\leq m-1,q_{ij}\neq q_{i,j+1}\right\}$.

#### 2.3.2 Local segmentation with CBS

We next perform local segmentation independently for each cell using the well-established Circular Binary Segmentation (CBS) (Olshen *et al.* 2004). CBS works by recursively splitting chromosomes into segments with maximal population means and using a t-statistic to test for significance. By default, the *P*-value is set to 0.05. Details on running CBS appear in Supplementary Note S1.3.

#### 2.3.3 Ensemble segmentation

Our ensemble method is designed to identify a final set of breakpoints for each cell by filtering the candidate GMM breakpoints based on global frequency, or number of cells which share the breakpoint, but relax the filtering if breakpoints overlap with the CBS results. Specifically, we add to the final set any candidate GMM breakpoint which is shared across *t* cells. To counterbalance the restrictive filtering, for each cell independently we also include any breakpoint present in both the candidate GMM and CBS breakpoints sets, regardless of its global frequency.

### 2.4 Ploidy estimation and inferring allele-specific copy numbers

SEACON jointly estimates cell ploidy and allele-specific copy numbers of each segment using the components derived with the GMM. For cell *i*, we first assign each segment *z* to the component with minimum euclidean distance in the feature space. Specifically, the component assigned to *z* is given by

(6) $$q_{z}=\underset{k}{\text{argmin}}\sqrt{{\left({\hat{X}}_{z}-{\hat{X}}_{k}\right)}^{2}+{\left({\hat{Y}}_{z}-{\hat{Y}}_{k}\right)}^{2}},$$

where ${\hat{X}}_{z}$ and ${\hat{Y}}_{z}$ to denote the RDR and mBAF of *z*. We define the function $h_{k}(z)$ for each component *k* such that $h_{k}(z)=1$ if segment *z* is assigned to *k*, and $h_{k}(z)=0$ otherwise. We wish to find the copy a number state $\left\{c_{k}^{\prime },c_{k}^{\prime \prime }\right\}$ for each component *k* with highest likelihood with respect to the expected component means defined previously. Let Ω be the set of all allele-specific copy number states with total copy number less than a user-defined threshold. If *p_i_* is known, we select each $\{c_{k}^{\prime },c_{k}^{\prime \prime }\}\in \Omega$ to be that which maximizes the following likelihood:

(7) $$L(p_{i};Z_{i})=\sum_{k}\sum_{z\in Z_{i}}h_{k}(z)\sum_{j\in z}(\ln\mathrm{Pr}(V_{ij}|c_{k}^{\prime },c_{k}^{\prime \prime },p_{i},\Sigma_{k})).$$

The copy numbers estimated from Equation (7) require the ploidy *p_i_* of each cell *i*. Ideally this is obtained through additional wet lab experiments, e.g. through fluorescence-activated cell-sorting (Navin *et al.* 2011). In most cases however, statistical estimation of *p_i_* is required, although this is challenging due to confounding factors such as whole-genome duplication (WGD). Many existing methods which compute total copy number infer *p_i_* from RDRs through a numerical optimization approach that minimizes the sum-of-squares error between the scaled copy number estimates after ploidy adjustment and their rounded integer values (Garvin *et al.* 2015). However, this may result in total copy numbers that contradict the degree of allelic imbalance. This shortcoming was addressed in CHISEL though an approach that restricts candidate values of *p_i_* to those that result in “balanced” bins (those with BAF ≈0.5) having a total copy number with no allelic imbalance. The value of *p_i_* is then chosen from the set of candidates using a BIC. One issue with this approach is that an equal weight is given to both RDR and BAF measurements, which may not be the case. To address this concern, we modify the approach of CHISEL to instead use a weighted BIC with weights derived from the typical sum-of-squares error computed over the RDRs. This assigns a greater impact to the RDR measurements while still incorporating the BAFs. On both real and simulated data, we show that this modified approach achieves more accurate ploidy estimates than that of CHISEL.

In detail, for each cell *i* we first identify the highest density component *k* with mBAF ≈0.5. Let ${\hat{X}}_{k}$ be the mean RDR of *k*. The set of candidate ploidies is computed as $\left\{\frac{2^{w+1}}{{\hat{X}}_{k}}\right\}$ for the number of WGDs w∈{0,1,…} up to a user-defined maximum. Then, *p_i_* is selected by minimizing the following weighted BIC

(8) $$p_{i}=\underset{p_{i}^{\prime }}{\text{argmin}}\phi (p_{i}^{\prime })\ln(m)|\Omega |-2L(p_{i}^{\prime };Z_{i}),$$

where ϕ is the standard sum-of-squares error for computing ploidy from only RDRs (Supplementary Note S1.4).

### 2.5 Simulations

We comprehensively evaluate SEACON in comparison to existing state-of-the-art methods on simulated datasets covering a wide range of experimental conditions. The simulated datasets used in our study were generated with the recently developed simulator CNAsim (Weiner and Bansal 2023). The CNAsim model is designed for evaluating single-cell CNA profiling and can be summarized in four main steps: (i) construct a cell-lineage tree with divergent subclonal populations; (ii) evolve an initially healthy diploid genome along the branches of the tree with CNAs; (iii) compile ground truth copy numbers for each cell and assemble complete sequences from the mutated genomes; (iv) generate synthetic scDNA-seq reads from the sequences. The CNAsim model also has several key features which are important in the context of this work. First, CNAs do not occur precisely along the breakpoints of predefined bins but instead are simulated at a much finer resolution than the bin size. Second, sequencing reads are sampled from two distinct reference haplotype sequences, thus enabling an end-to-end evaluation of allele-specific copy number profiling. To the best of our knowledge, there does not yet exist a benchmark for single-cell allele-specific CNA profiling from scDNA-seq reads.

We generated a total of 36 datasets which are broadly classified into 2 categories: direct simulation of read count and BAF matrices, and simulation of whole-genome scDNA-seq reads. The former consists of datasets C1–C6 and involves directly drawing read counts and BAFs for each bin based on the ground truth copy number state instead of deriving these values from sequencing reads. These datasets were used to evaluate the merits of the ensemble segmentation approach.

The latter category contains the majority of the simulated datasets, A1–A16 and B1–C15, and consist of raw fastq files for each cell. All datasets were generated using two custom whole-genome haploid reference sequences with known SNPs, enabling both read count and BAF estimation. The datasets explore various experimental conditions, with the main parameters being number of cells ∈{100,500,1000,5000}, coverage ∈{0.02X,0.05X,0.1X,0.25X,0.5X}, bin size ∈{500kbp,1Mbp,5Mbp}, and ploidy ∈{hypodiploid, diploid, triploid, tetraploid}.

A complete description of the simulation pipeline including a detailed explanation of parameters and their values is in Supplementary Section S3 and Supplementary Tables S1–S3.

### 2.6 Evaluation

We compare SEACON with three allele/haplotype-specific copy number methods in Alleloscope (Wu *et al.* 2021), SIGNALS (Funnell *et al.* 2022), and CHISEL (Zaccaria and Raphael 2021), as well as four total copy number methods in SCOPE (Wang *et al.* 2020), SeCNV (Ruohan *et al.* 2022), SCONCE (Hui and Nielsen 2022), and HMMCopy (Shah *et al.* 2006). As each method has a different data preprocessing strategy, the number of cells and bins in the predicted copy number profiles may vary. For consistency, we extract a common set of cells and bins present in the outputs of all methods for use in the evaluations. For each dataset, methods which fail to generate results for at least 50% of the cells and bins are excluded. Further details and parameters used to run the methods are listed in Supplementary Section S4.

To compare accuracy, we evaluate each method in terms of absolute copy number estimation, ploidy estimation, and breakpoint detection. Absolute copy number accuracy was measured using the log-scaled sum-of-squares error (LSSE) between the true and estimated total copy numbers for each bin of every cell. For SEACON and CHISEL, we also measure allele-specific copy number inference equivalently. We specify “total-LSSE” and “allele-LSSE” to be for total copy numbers and allele-specific copy numbers, respectively. As this metric is susceptible to being skewed from incorrect ploidy estimation, we also consider an adjusted total-LSSE where inferred total copy numbers are scaled so that the estimated cell ploidies are equal to the ground truth. To evaluate breakpoint detection, we compute the precision, recall, and F1-score of the predicted breakpoints for a tumor cell such that a predicted breakpoint is correct if it is within *l* bins of a true breakpoint. Because inferring many false positives will artificially increase precision for values *l *>* *1, each true breakpoint can validate exactly predicted breakpoint. We maximize the number of validated predicted breakpoints by formulating the true and predicted sets as a bipartite graph and computing a maximum matching.

## 3 Results

We run SEACON on the simulated datasets to assess its ability to infer absolute copy numbers, estimate ploidy, and detect breakpoints. We set the minimum and maximum number of components to 8 and 25, respectively, and use a merge distance threshold *δ* of 0.1. For segmentation, we evaluate SEACON with and without breakpoint filtering by setting the global frequency threshold *t* to 5 and 1 cells.

### 3.1 Segmentation performance

We first explore the breakpoint filtering and ensemble segmentation procedure of SEACON to better understand each part. We applied the individual segmentation steps to the six datasets with directly simulated read counts and BAFs, C1–C6, which represent all combinations of low and high noise over three different coverage settings.

Supplementary Table S4 shows the accuracy of the GMM breakpoints when they are shared across *t* cells. As expected, using higher values for *t* results in higher precision but lower recall. In the low noise setting, the baseline performance for *t *=* *1 is sufficiently high that this trade-off has a mostly negative effective due to over-filtering. However, the benefits of the breakpoint filter become apparent in the high noise setting where the increase in precision is significant.

We next evaluate the GMM and CBS components in isolation and compared them to the ensemble approach of SEACON (Supplementary Table S5). Overall, CBS tends to have higher precision but lower recall compared to the GMM. When used together, the ensemble approach has a much higher precision than either CBS or GMM alone, while the relaxation of the breakpoint filtering with CBS greatly negates the loss in recall.

### 3.2 Absolute copy number accuracy

**Figure 2.**
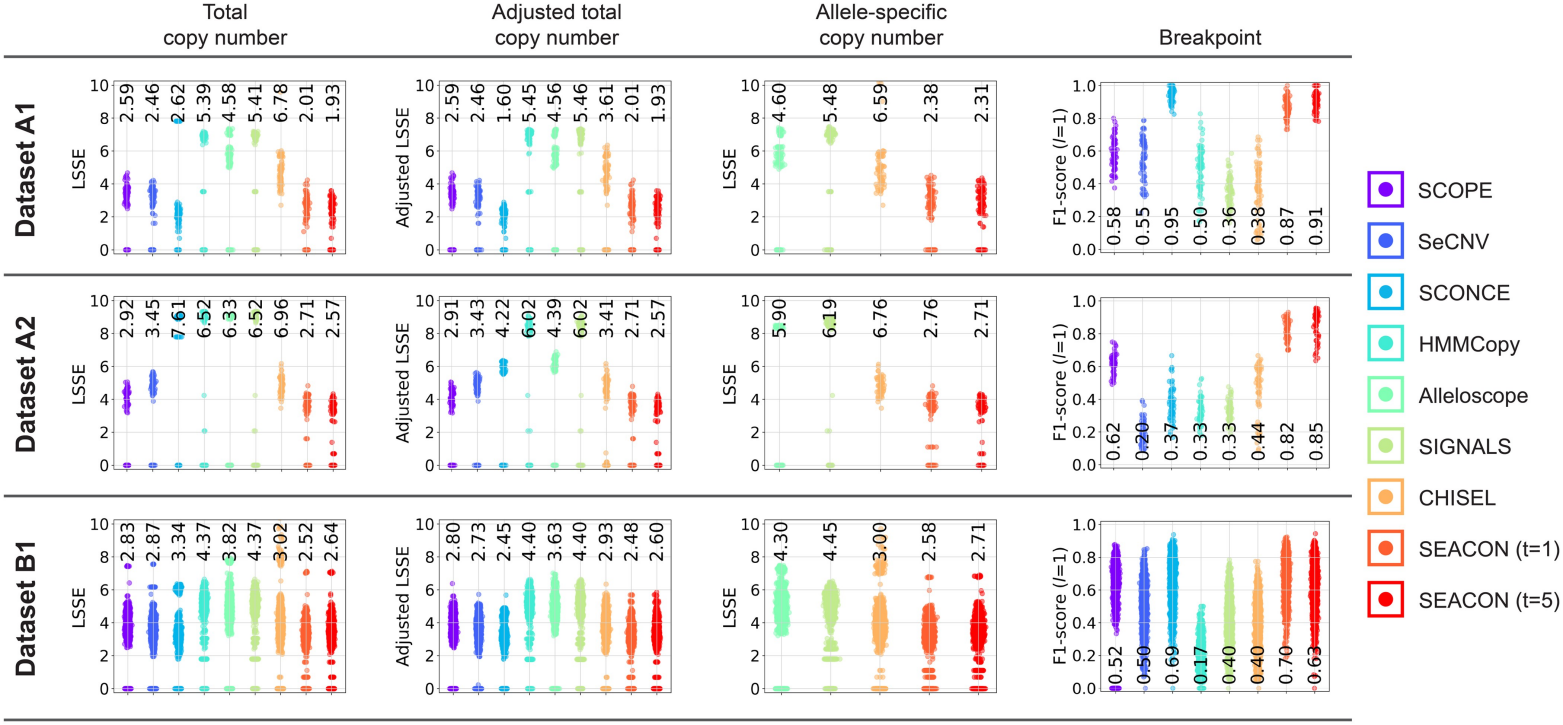
Various accuracy results of the copy number detection algorithms on three simulation sets: A1 (top row, 100 cells, 0.1X coverage, diploid), A2 (middle row, 100 cells, 0.1X coverage, tetraploid), and B1 (bottom row, 5000 cells, 0.02X coverage, diploid). See Supplementary Section S3 for details. The first column shows the log-scaled sum-of-squares errors (LSSE) between the estimated and ground truth total copy numbers. The second column shows the LSSE over total copy numbers adjusted for ploidy estimation error. The third column shows the LSSE between the estimated and ground truth allele-specific copy numbers. The fourth column shows the F1-score between the predicted and true breakpoints, where predicted breakpoints are correct if they fall within *l *=* *1 bins of a true breakpoint. Each dot represents the mean value of one cell, and the mean value across cells is printed inline with each method.

We next describe the absolute copy number accuracy of each method on the simulated whole-genome scDNA-seq datasets. For brevity, we show representative datasets A1, A2, and B1 in Fig. 2, and the remainder of the datasets are shown in Supplementary Figs S4–S9. Both A1 and A2 have 100 cells, 25% of which are normal, 0.1X coverage, and 1Mbp bins, but differ in ploidy (A1 has diploid tumor cells whereas A2 is tetraploid). B1 is larger in scale with 5k cells, 0.02X coverage, 5 Mb bins, and diploid tumor cells. Heatmaps of the genome-wide profiles from each method appear in Supplementary Fig. S2.

Overall, SEACON consistently outperforms all other methods in terms of total copy number accuracy across the vast majority of datasets, and especially outperforms Alleloscope, SIGNALS, and CHISEL in terms of both total and allele-specific copy number accuracy. On dataset A1 (uses a bin size of 5 Mbp), SEACON achieves a total-LSSE of 2.01 and 1.93 for *t *=* *1 and *t *=* *5, respectively, and slightly worse values for allele-LSSE at 2.38 and 2.31. By comparison, the total copy number methods SCOPE, SeCNV, SCONCE, and HMMCopy have a total-LSSE of 2.59, 2.46, 2.62, and 5.39, respectively, and the allele-specific methods Alleloscope, SIGNALS, and CHISEL have an allele-LSSE of 4.6, 5.48, and 6.59, respectively. We find that the three allele-specific methods besides SEACON generally struggle with ploidy estimation, partially leading to the relatively high values.

On dataset A2, which has near tetraploid tumor cells, all methods perform strictly worse, although SEACON (*t *=* *5) is again the best performing method with a total- and allele-LSSE of 2.57 and 2.71, respectively. The decrease in performance is likely due to difficulties in identifying the exact copy number state of high amplitude bins, and can be observed in other datasets with high ploidy (see A6, B5–B6, B10–13, Supplementary Figs S4 and S7–S9). On dataset B1, which has 5k cells and uses larger bins, SEACON achieves a total-LSSE of 2.52 and 2.64 for *t *=* *1 and *t *=* *5, respectively, and allele-LSSE values of 2.58 and 2.71. We find that for datasets that use large bin sizes and/or sufficiently high total coverage, setting *t *>* *1 only decreases performance, likely due to filtering out correct but low frequency breakpoints. In either case, both settings of SEACON are better than all other methods in both categories.

### 3.3 Adjusted copy number accuracy

To account for the effect of ploidy misestimation in the observed differences in copy number accuracy, we also consider the adjusted LSSE of total copy numbers. It is expected that methods which accurately predict ploidy will closely match their unadjusted counterparts. For example, on dataset A1 SCOPE, SeCNV, HMMCopy, Alleloscope, SIGNALS, and SEACON all have nearly identical LSSE and adjusted-LSSE values, whereas SCONCE and CHISEL both have substantially lower adjusted-LSSE. These observations are reflected by the fraction of the genome with correct inferences, which shows these two methods have some cells with no correct inferences (Supplementary Fig. S3).

We find that SEACON accurately estimates ploidy and thus shows minimal change in unadjusted versus adjusted-LSSE on the majority of datasets. The only exceptions are datasets C6 and C7 (Supplementary Fig. S7), which have extreme coverage nonuniformity and affect other methods similarly, and dataset B8 (Supplementary Fig. S8) which has hypodiploid tumor cells and proved challenging for all methods except for CHISEL. Outside of these datasets, SEACON achieves the lowest or a comparable adjusted-LSSE compared to the best performing total copy number method, and a lower adjusted-LSSE than all allele-specific methods.

### 3.4 Breakpoint accuracy

We next evaluate the accuracy of each method without factoring in the actual copy number estimation by comparing the breakpoints between the predicted and true copy number segments. We find that SEACON achieves the highest F1-score compared to the other methods in 2/3 of the datasets and maintains a competitive F1-score with the best performing method on the remaining 1/3. The conditions where SEACON achieves higher accuracy tend to be over larger ploidies or bins with sufficient total coverage. For example, on tetraploid dataset A2 SEACON (*t *=* *5) has an F1-score of 0.85 compared to the next best method SCOPE at 0.62 and the next best allele-specific method CHISEL at 0.38 (see also datasets B5–B6, B8–B13, Supplementary Figs S7–S9). On dataset B1, SEACON (*t *=* *1) has the top F1-score of 0.7 followed closely by SCONCE at 0.69, while SIGNALS and CHISEL are both at 0.4 (see also datasets A4–A9, B2–B4, Supplementary Figs S4, S5, and S7). Datasets where SEACON does not achieve the highest F1-score, such as in dataset A1, tend to have low total coverage relative to bin size (see datasets A3, A10–A11, A16, B14–B15, Supplementary Figs S4–S6 and S9), but is nearly always the second-best performing method by a significant margin.

We also examine the individual precision and recall values of each method and the example heatmaps of dataset A1 to gain further insight into the general segmentation behavior of each method (Supplementary Tables S6–S8, Supplementary Fig. S2). As reflected by the reported F1 scores, both SEACON and SCONCE are highly similar to the ground truth and capture many of the smaller localized breakpoints. Ignoring ploidy, SCOPE, SeCNV, HMMCopy, and CHISEL are more conservative in their predictions, accurately capturing major shifts but smoothing over many small CNAs. SIGNALS also finds the major shifts, but returns numerous erroneous breakpoints.

Lastly, we investigated if the segmentation results of CHISEL could be improved using the ensemble segmentation procedure in SEACON by swapping the GMM components with the inferred bin clusters of CHISEL (Supplementary Table S9). Just as with SEACON, we found that this improved the performance of CHISEL on datasets A1 and A2 but was slightly disadvantageous on dataset B1, suggesting that the breakpoint filtering heuristic will generally lead to better results but that SEACON remains the more accurate method.

### 3.5 Analysis of scDNA-seq data of breast cancer patients with FACS ploidy validation

We applied SEACON to two single-cell DNA-seq datasets from triple-negative breast cancer patients (denoted T16 and T10) Navin *et al.* (2011). Fluorescent-activated cell sorting (FACS) was used to gate 100 single cells from each patient based on observed ploidy distributions. In patient T16, 52 cells were sampled from the primary tumor and 48 cells from a paired liver metastasis, with FACS showing a total of 62 diploid cells (ploidy ≈2) and 38 aneuploid cells (ploidy ≈4.1). In patient T10, FACS revealed four ploidy distributions: 47 diploid/pseudo-diploids (ploidy ≈2), 24 hypodiploids (ploidy ≈1.7), 25 A-type aneuploids (ploidy ≈3), and 4 B-type aneuploids (≈3.3).

Raw reads in the form of fastq files were obtained from the NCBI Sequence Read Archive (SRA018951). We aligned the raw reads to the hg38 reference genome and used 1Mbp bin lengths.

**Figure 3.**
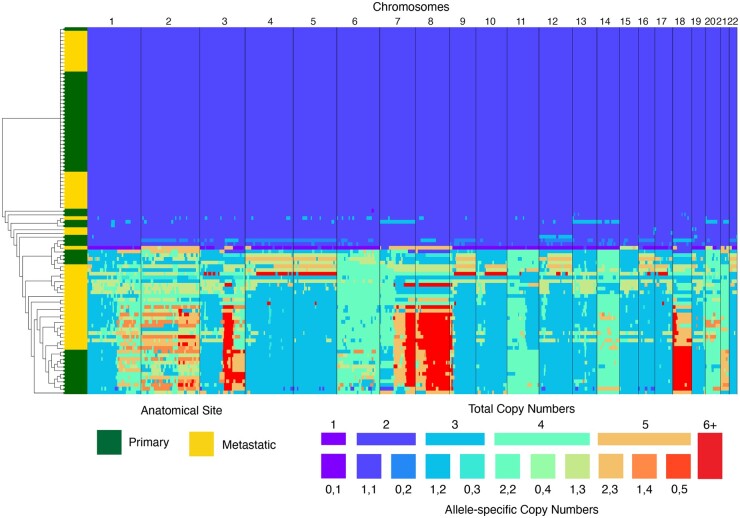
Whole-genome allele-specific copy number profiles of patient T16 inferred by SEACON. The leftmost column shows the anatomical location from which the cell was sampled, and a cell-lineage tree was constructed under a model of CNA evolution. Consistent with FACS analysis, the results show a large population of healthy diploid cells, and a population of near-tetraploid tumor cells.

Figure 3 and Supplementary Fig. S10 show heatmaps of the estimated allele-specific copy number profiles for patients T16 and T10, respectively. We constructed cell-lineage trees over the populations using pairwise distances between the cells computed under a model of CNA evolution (Schwarz *et al.* 2014) (see Supplementary Note S1.5). Consistent with previously reported results on T16 (Navin *et al.* 2011, Ruohan *et al.* 2022), the aneuploid cells show evidence for having undergone many shared large-scale aberrations, likely a WGD early on in the tumor lifespan followed by an abundance of deletions on chromosomes 4–5, 9–10, 12–13, 15–17, 19, and 22. The early WGD is supported by inferred ancestral genomes in the cell-lineage tree, which places the WGD along the branch leading into the common ancestor of all tumor cells. The majority of these cells represent a monogenetic population contained within a large clade with well-separated components from both anatomical sites, and feature repetitive amplifications on chromosomes 8, 18, 3q, and 7q. On patient T10, the grouping of ploidy fractions into distinct clades and their order of divergence from the normal cells is consistent with the previous reported results (Navin *et al.* 2011).

To evaluate ploidy detection, we computed the pearson correlation coefficient (PCC) and the root-mean-square error (RMSE) between the estimated cell ploidies and those detected by FACS. On patient T16, SEACON was highly consistent with the FACS analysis with a PCC and RMSE of 0.977 and 0.242, respectively. SCOPE had a relatively high PCC at 0.921 but a RMSE of 0.734, suggesting that the overall copy numbers signals were detected reasonably well but the ploidies were estimated poorly. The PCC and RMSE of CHISEL were 0.607 and 1.088, respectively, highlighting the importance of down-weighting noisy BAFs. The higher consistency of SEACON over SCOPE can be partially attributed to the integration of allele frequency signals in the estimation through the ruling out of contradictory copy number states; e.g. on chromosomes 5 and 6, which have mean BAFs of 0.262 and 0.386, respectively, SEACON returned consensus aneuploid copy number states of {1, 2} and {2, 2} while SCOPE, unaware of the BAFs, returned total copy numbers 2 and 3, respectively (see Supplementary Figs S11 and S12).

On patient T10, the respective PCC and RMSE values were 0.879 and 0.589 for SEACON, 0.089 and 1.492 for CHISEL, and 0.974 and 0.122 for SCOPE. We found that SEACON correctly estimated the ploidy of the hypodiploid cells but misestimated that of the aneuploid cells, which in isolation had RMSEs of 0.135 and 1.103, respectively. The incosistency of SEACON with the FACS analysis is due to the level of noise in the BAF measurements obfuscating any meaningful signal of allelic imbalance (Supplementary Fig. S13). The higher noise rate in T10 compared to T16 is a consequence of the sequencing coverage; T10 has an average of 6 616 889 reads per cell compared to 10 766 075 for T16. Overall, this dataset highlights a limitation of integrating BAFs into cell ploidy estimation.

### 3.6 Analysis of low-coverage scDNA-seq data of triple-negative breast cancer patient

We also evaluate SEACON on a publicly available 10x Genomics scDNA-seq dataset consisting of 2075 cells from a frozen triple-negative ductal carcinoma. The 2075 cells represent a single anatomical sector (sector E) from a larger 5-sector dataset sampled from the same tissue, totaling ≈10k cells. In a previous study, allele-specific CNA profiles generated with CHISEL (Zaccaria and Raphael 2021) identified one diploid clone (labelled I) with 390 cells and five main tumor clones (labelled II-VI) with 168, 58, 20, 782, and 30 cells, respectively, for a total of 1448 cells. The remaining 627 cells were removed from analysis due to clustering poorly with the population, however a re-evaluation of the removed cells with SEACON suggests that >25% belong to the six main clones (see Supplementary Section S2.2).

Previous computed CHISEL calls and BAF estimates over 5Mbp bin sizes were obtained from the chisel data repository (https://github.com/raphael-group/chisel-data). The BAFs were originally computed across the ≈10 000 cells from the same tissue. Despite low sequencing coverage of between 0.02X and 0.05X per individual cell, the BAF estimation routine of CHISEL was shown to increase in accuracy with an increase in total sequencing coverage across cells. As shown from our simulations using large bins and accurate BAFs, the CHISEL calls are considerably more accurate. Thus, we expect to observe highly similar results from both methods under these ideal conditions.

Supplementary Figure S14a shows the heatmap of the allele-specific copy numbers derived by SEACON over 5 Mb bin sizes for original clones I–VI. We found that the results SEACON remained consistent for merge distance thresholds δ∈{0,0.05,0.1,0.15}, indicating accurate BAF measurements. We compared the results of SEACON to those of CHISEL, and cells were sorted according to the CHISEL clone labels. A heatmap of the CHISEL results are shown in Supplementary Fig. S14b. As expected, the results of both methods are highly consistent with one another, with all but 13 of the 1448 cells clearly resembling the CHISEL clone label, and an identical set of divergent chromosomal CNAs that differentiate each clone. Furthermore, the cell ploidy estimates were highly consistent between the two methods with a PCC of 0.998, with strong evidence for WGD. The main difference is that SEACON shows frequent low-frequency focal CNAs, while CHISEL tends to smooth the small segments.

## 4 Discussion

CNAs are key drivers of cancer progression and their accurate detection at the single-cell level has motivated the development of many computational methods. This includes detecting allele-specific copy numbers, which are important for revealing rare and complex CNAs. Recently, the CHISEL algorithm (Zaccaria and Raphael 2021) enabled the computation of BAFs in low coverage scDNA-seq data, but further model development toward detecting allele-specific CNAs has not been explored. In this work, we develop a framework for allele-specific CNA profiling, SEACON, which improves upon CHISEL through several novel features. This includes a strategy for tolerating noisy measurements through a post hoc weighted merging of GMM components and a weighted BIC for detecting ploidy using both read count and BAF signals. Additionally, SEACON implements an ensemble segmentation approach which combines global and local breakpoint detection paradigms. To the best of our knowledge, SEACON is the first method to explore an ensemble approach to segmentation in the single-cell setting.

We show that SEACON outperforms Alleloscope, SIGNALS, and CHISEL in every metric across nearly all simulated datasets, but particularly on smaller bin sizes. When conditions are more favorable to the design of CHISEL, namely the use of large bins and sufficient total coverage, the results become more comparable, however SEACON remains the top method. Compared to the total copy number methods, SEACON identifies both breakpoints and total copy numbers more accurately than SCOPE, SeCNV, and HMMCopy across nearly all simulated datasets, while either outperforming or achieving a performance on par with SCONCE, the best total copy number method. Our simulations also reveal the breakpoint detection patterns and the segmentation strategies of each method. In general, most methods are highly selective leading to high precision at the cost of recall, including SCOPE, SECNV, SCONCE, and HMMCopy, and to a lesser extent SIGNALS and CHISEL. SEACON achieves a much greater balance between precision and recall, but the breakpoint filtering offers control over this ratio which, depending on the bin resolution, can lead to overall better results.

There remain several limitations of SEACON and opportunities for future work. First, SEACON makes no methodological improvements toward estimating BAFs, but instead uses the procedure in CHISEL. On a related note, the accuracy of existing BAF estimation approaches have not been thoroughly evaluated, and the simulation pipeline used in this work could facilitate that. Second, our analysis of both real and simulated datasets demonstrated that SEACON begins to break down when the coverage is too low. In these cases, it is better to use an existing method for inferring total copy numbers, and a diagnostic program to determine this decision would be helpful. Third, while the down-weighting of BAF values during component merging can be an effective way to overcome noise, the strategy itself is rudimentary and may incorrectly merge components which represent two distinct copy number states but with similar expected mBAFs, e.g. {3, 4} versus {2, 5}. It may be interesting to take an opposite segmentation approach to SEACON, namely to first derive breakpoints from a more sensitive segmentation technique first, and then to cluster the segments rather than bins. However, this approach comes with its own downsides, e.g. it may miss breakpoints detectable only by BAFs. Fourth, SEACON does not leverage the evolutionary relationships between cells during inference. One straightforward idea is to filter breakpoints based on their frequency among closely related cells, rather than their global frequency across all cells in the sample. Fifth, SEACON could be extended to infer haplotype-specific copy numbers, which may reveal rare but interesting mirrored CNAs. Lastly, one may find results can improve if other segmentation algorithms are used within the SEACON framework instead of the GMM and/or CBS, particularly as novel approaches become available.

## Supplementary Material

btae506_Supplementary_Data

## Data Availability

An open-source implementation of SEACON and simulation scripts are freely available from https://github.com/NabaviLab/SEACON and https://doi.org/10.5281/zenodo.12727008.
