# Supplementary material for: Improved allele-specific single-cell copy number estimation in low-coverage DNA-sequencing
Source: Bioinformatics. 2024 Aug 12;40(8):btae506. doi: 10.1093/bioinformatics/btae506 (PMC11346770; doi:10.1093/bioinformatics/btae506)
Supplement: btae506_Supplementary_Data [file btae506_supplementary_data.pdf]

Supplementary Material

S1 Supplementary Notes

S1.1 Normal cell identification

Normal cells are identified from the raw read counts using the gini coefficient, a measure of inequality among the values of a frequency distribution.

For cell i, it's gini coefficient is computed as

$$g_i = \frac{\sum_{j=1}^{m} \sum_{k=1}^{m} |R_{ij} - R_{ik}|}{2m \sum_{j=1}^{m} R_{ij}} \tag{1}$$

Since tumor cells will have heterogenous read count signals, the distribution of gini coefficients is expected to contain a single peak for normal cells, and one or more peaks for tumor cells. Cells are assigned to one of k boxes that equally divide the interval $0 - 1$ based on their gini coefficient, with the value of a box being the number of cells assigned to it. Boxes are selected as peaks by comparing them to the value of their neighbors. SEACON selects the set of normal cells to be those assigned to any box in the first peak.

S1.2 Weighted component merging

After an initial number of components is selected using a BIC, SEACON adopts a post-hoc component merging to address over-clustering. There are two aspects of the feature space that may result in over-clustering. First, high variance BAF measurements may lead to multiple components representing the same copy number spread across the mBAF axis. In particular, while the quality of read counts rely mostly on sequence coverage and distribution, BAFs also require a high global coverage across all cells, which may not always be available. Second, the global distribution of bins to copy number states is extremely uneven when considering the whole genome. Regions which undergo extreme amplification exist but are rare, resulting in the vast majority of bins belonging to a small set of common states ({1,1}, {1,2}, {2,2}, ect...). As a result, the model may choose to partition high density components into two or more subcomponents. To correct for possible overclustering, SEACON refines the mixture of components by iteratively merging pairs of components whose means fall within a certain distance threshold. In particular, we define the distance between a and b to be

$$d(a, b) = \sqrt{\hat{X}_a - \hat{X}_b)^2 + (\hat{Y}_a - \hat{Y}_b)^2}, \tag{2}$$

where $\hat{X}_a$ and $\hat{Y}_a$ (resp. $\hat{X}_b$ and $\hat{Y}_b$) are the respective mean RDR and BAF of a (resp. b). For some threshold δ, if $d(a, b) < \delta$, we merge components a, b as follows:

$$\mu_{ab} = \frac{\psi_a \mu_a + \psi_b \mu_b}{\psi_a + \psi_b}, \text{ and} \tag{3}$$

$$\Sigma_{ab} = \frac{\psi_a^2 \Sigma_a + \psi_b^2 \Sigma_b + (\psi_a \mu_a + \psi_b \mu_b)(\psi_a \mu_a + \psi_b \mu_b)^T}{(\psi_a + \psi_b)^2} - \mu_{ab} \mu_{ab}^\top. \tag{4}$$

S1.3 Circular binary segmentation

To perform the local segmentation on each sample's read counts, we use Circular Binary Segmentation (CBS) [10]. Given m bins over cell i, denoted as $x_i, i = 1, ..., m$, CBS method converts read count data regions of equal copy number. The CBS method treats the read count data as a circle and looks for two points that divide the circle into two arcs with the maximum difference in their population means of the read count data. Let's denote the partial sum on one cell as $S_i = x_1 + ... + x_j, 1 \leq j \leq n$. The likelihood test statistic for the hypothesis that the arc from $j + 1$ to k and the other arc have different population means is given by:

$$Z_{jk} = \{1/(k - j) + 1/(m - k + j)\}^{-1/2} \{(S_k - S_j)/(k - j) - (S_m - S_k + S_j)/(m - k + j)\}.$$

CBS finds $Z_C = \max_{1 \leq j < k \leq m} |Z_{jk}|$. Since the test statistics assumes the normality of the data, CBS uses permutation on the original data to generalize the algorithm to non-normal data.

S1.4 Ploidy estimation weights

Many existing methods which infer total copy number estimate ploidy through a numerical optimization approach using the RDRs [6,11,17]. This approach works by minimizing the sum-of-squares error (SSE) between the RDRs adjusted for ploidy and their rounded integer values. For some candidate ploidy p'_i, the sum-of-squares error is computed as

$$f(p'_i) = \sum_{j=1}^{m} \Big((p'_i \hat{X}_{ij}) - (\lfloor p'_i \hat{X}_{ij} \rceil) \Big)^2, \tag{5}$$

where $p'_i \hat{X}_{ij}$ is the scaled total copy number and $\lfloor p'_i \hat{X}_{ij} \rceil$ is the scaled total copy number rounded to the closest integer. The ploidy is then chosen to be that which minimizes f among candidates $\{1.5, 1.55, 1.6, ..., 5.5\}$ or other bounds.

The weight function ϕ used to compute ploidies in Equation 8 uses the same numerical optimization approach. Recall that k is the highest density component with mBAF ≈ 0.5 and $\hat{X}_k$ the mean RDR of k. Each candidate ploidy p'_i for cell i is from the set $\{\frac{2^{w+1}}{\hat{X}_k}\}$ where w is the number of whole-genome duplications (WGDs). For example, if $\hat{X}_k = 1$ and the max number of WGDs considered is 2, then the candidates would be $\{2, 4, 8\}$. By default, we directly set $\phi(p'_i) = f(p'_i)$. However, this may be overly restrictive if the candidate values deviate slightly from the true ploidy, which is expected. To address this, we also consider setting $\phi(p'_i)$ to be the minimum SSE for values in a range centered around p'_i, for example $\{p'_i - 0.2, p'_i - 0.19, p'_i - 0.18, ..., p'_i + 0.2\}$.

S1.5 Constructing cell-lineage trees

To construct a cell-lineage tree over a population of cells, we first compute pairwise distances between each cell from their allele-specific copy number profiles under the copy number transformation (CNT) model [12]. Under this model, the copy number distance (CND) between two profiles is defined as the minimum number of segmental amplifications and deletions needed to transform one profile into the other. Because CNAs occur on chromosomes independently, the total distance between two cells is the sum over the CNDs of each chromosome. Individual chromosome pairs were distinguished by trivially phasing the allele-specific copy numbers using a major/minor haplotype configuration, which works reasonably well in most cases [8].

Given the distance matrix, the balanced Minimum-Evolution algorithm [4] is used to build the topology from the computed distances, which works by searching for a tree topology with minimum branch lengths. The result is an unrooted binary tree where leaves correspond to observed cells. The tree is rooted by setting the least-common ancestor (LCA) of the normal cells as the outgroup. The normal cell LCA was chosen to be the clade with the highest proportion of normal cells among clades where $\geq 95\%$ of normal cells are descendants. To infer ancestral states, we use MEDICC2 [8] by providing the tree topology and copy number profiles at the leaves. MEDICC2 also returns CNA events that occur along each branch in the tree.

S2 Supplementary Results

S2.1 Quality control

Following standard practices, after alignment and read filtering, SEACON masks bins with extreme GC content or poor mappability. The autosomes of the hg38 reference genome total to $\approx 2.8 \times 10^8$ base pairs, and partitioning the genome into bins of size 5Mbp and 1Mbp results in a total of 587 and 2887 bins, respectively. By default, SEACON masks bins with GC content < 0.2 or > 0.8 or mappability < 0.9. For 5Mbp and 1Mbp bin sizes, this process removes 62 and 335 bins, respectively, which is equivalent to 10.6% and 11.6% of the genome being labelled as "blacklisted". For the remaining bins, SEACON normalizes the read counts using normal cells identified in the sample, which implicitly corrects for GC content bias, mappability bias, and other latent factors. If no normal cells are available, read counts are normalized for GC content bias and mappability bias similar to [5].

We investigated if cancer-related regions of interest are contained within bins which pass the filtering of SEACON. A set of cancer-related genes was obtained from the COSMIC Cancer Gene Consensus [14] which totalled to 707 across the autosomes of hg38. Under the default GC content and mappability thresholds used to mask bins, 671 and 666 genes remain in the filtered sets of 5Mbp bins and 1Mbp bins, respectively, or equivalently 95% and 94%.

S2.2 10x dataset clone analysis

To compare the results of SEACON with a previous analysis with CHISEL [20] on the 2075 cells from the 10x genomics dataset, we grouped the cells into six clones (named I-VI) according to the originally reported cell clone labels. These clones were derived using hierarchical clustering based on the inferred CNA profiles, and it was shown that clones could be recovered if they had ≈ 10 cells or more. The clones consist of one diploid subpopulation (clone I with 390 cells) and five main tumor subpopulations (clones II-VI with 168, 58, 20, 782 and 30 cells, respectively). The remaining 627 cells of the original 2075 not belonging to any clone were labeled as noisy and removed from downstream analysis. It was shown that these cells cluster poorly with the other cells, and it was suggested that this noise was caused by cells being in the S-phase of the cell cycle, having abnormally low reads, or doublets.

Using the CNA profiles generated with SEACON, we investigated whether any of the 627 ungrouped cells are "rescued" by showing evidence for belonging to a significant clonal population. First, we quantify the relatedness of the existing clones by computing the average copy number distance (CND) [12] between each cell in the clone with the clone's consensus CNA profile (see Supplemental Section S1.5). For clones I-VI, the average CNDs were $5.577, 21.512, 31.431, 31.9, 27.4$, and 20.5, respectively. Next, for each ungrouped cell, we compute the CND between it and the consensus profile of each clone. For any clone I-VI, if this distance is less than the average clone CND, then we consider this cell to be "rescued" (note that we also ensure the cell ploidy is compatible with the clone ploidy, as WGDs are poorly reflected in the CND). Interestingly, we found this to be the case for 162/627 or 26% of the ungrouped cells, and suggests that SEACON is able to produce more accurate results for noisy cells compared to CHISEL.

Lastly, we checked whether there existed clonal populations within the remaining 465 ungrouped cells which were not reported at all. We applied hierarchical clustering on precomputed pairwise CNDs between the cells, and set the threshold on the maximum average clone CND (defined above) before cutting the dendrogram to be 32 as that is the maximum of clones I-VI. This resulted in clusters of size no greater than 2, confirming that the remaining ungrouped cells cluster extremely poorly with the rest of the population.

S3 Simulation

All simulated datasets in this study were generated using the simulation tool CNAsim [18]. CNAsim is able to generate ground truth copy number profiles (CNPs) and allows synthetic sequencing reads to be sampled from two separate reference genomes, one for each haplotype, making it suitable for benchmarking total and allele-specific CNA detection methods. We generate two types of datasets with CNAsim. First, we directly simulate read counts and BAFs from the ground truth copy numbers, bypassing the generation of sequencing reads and thus the preprocessing steps of CNA detection. We created six of these datasets labelled C1-C6. Second, we generate synthetic sequencing reads from the mutated tumor genomes, enabling an end-to-end evaluation of CNA detection algorithms. We created 31 of these datasets labelled A1-A16 and B1-B15.

In this section, we first describe how CNAsim was overall used and the parameters which were consistent across all datasets. For full details on the CNAsim model, we refer the reader to [18]. We next describe in detail the complete pipelines used to generate both types of datasets and the important parameters used. For a condensed list of the simulated datasets and their key parameters, see Supplementary Tables S2 and S3.

S3.1 Overall usage of CNAsim and fixed parameters

CNAsim simulates a cell-lineage tree describing the evolutionary history of a tumor population from which n cells are observed. Each cell, both ancestral and observed, is represented by a genome consisting of K chromosomes, where each chromosome is a vector of M-length regions. The region length essentially dictates the resolution at which CNAs are simulated. If the region length is equal to the bin length, then CNA detection is expected to become much easier as CNAs occur exactly along the breakpoints between bins, while smaller region lengths make inference more challenging. We use a default region length of 10kbp, but we also explore region lengths of 1Mbp and 5Mbp. Of the n observed cells, a fraction of them are selected to be normal healthy cells which do not undergo any CNAs. Most datasets have $0.25n$ normal cells, but we also explore $0.1n$ and $0.4n$. Additionally, a fixed number of ancestral cells

are selected to become subclonal founders. These cells undergo larger chromosomal events compared to other cells, thereby further separating their lineage from the rest of the population. The number of subclones was scaled depending on the number of observed cells (3 subclones for 100 cells, 8 for 500, and 12 for 1000, 24 for 5000).

Focal CNAs occur along each edge of the tree, the number of which is the default mean of 2 per edge. We used the simulator defaults for event properties, namely a mean length of 5 Mbp and equal probability of amplification vs deletion. A WGD can be included into the founder tumor cell with a toggle to create higher ploidy populations, but is toggled off by default. Chromosome and chromosome-arm deletions and duplications can occur in the founder cell, superclones founders and subclones founders. The mean number of these events was 3 for each edge, though we also explore setting this number to 5 and 7. We set the probability of a chromosomal event occurring on a chromosome arm vs the whole chromosome to be 0.75. In the absence of a WGD, the duplication vs deletion rate was equal to keep the tumor ploidies near diploid. If a WGD is included, the duplication rate was set to 0.25. This results in ploidies of approximately 2 and 3.5, respectively. We also explore setting the duplication rate to 0.5 and 0.75 with WGD to simulate ploidies of approximately 4 and 4.5, respectively.

After evolving the genomes along the tree, default parameters we used to generate the ground truth copy number profiles and, for the latter group of datasets, generate synthetic sequencing reads. The size of the cell genomes used in the simulation are as follows: For datasets C1-C6 (directly simulated read counts and BAFs), we set the number of chromosomes to 10 and each a fixed length of 100 Mbp. For the remaining datasets A1-A16 and B1-B15 (synthetic sequencing reads), we used the 22 autosomes and their corresponding lengths derived from the hg38 reference genome.

S3.2 Direct read count and BAF simulations

To bypass preprocessing and evaluate the segmentation potential of SEACON directly, we consider a simulation model where the read count and BAF measurements of each bin are simulated directly. We begin with the ground truth CNPs of each cell generated with CNAsim. We assume that the read counts follow a Poisson distribution $Poiss(s*(c'+c''))$, where $\{c', c''\}$ is the allele-specific copy number state and s is the scale factor which corresponds to sequence coverage. We subject the read counts to noise by multiplying with Gaussian noise $\mathcal{N}(1, \sigma_r)$, where σ_r is the read count noise rate. BAFs are simply set to $\frac{\min c', c''}{c'+c''}$ multiplied by a draw from a Gaussian $\mathcal{N}(1, \sigma_b)$, where σ_b is the BAF noise rate.

We evaluated a low noise setting with $\sigma_r = 0$ and $\sigma_b = 0.01$ and a high noise setting with $\sigma_r = 0.1$ and $\sigma_b = 0.03$. Note that even with $\sigma_r = 0$, there is inherent noise from the Poisson distribution. For both noise settings, we evaluated three scale factors $s \in \{100, 300, 600\}$. These combinations comprise the six datasets D1-D6. See Supplementary Table S1 for a detailed list of the parameter values.

S3.3 Synthetic DNA sequencing reads simulations

For an end-to-end evaluation of the CNA detection pipeline, we use CNAsim to generate raw sequencing reads in the form of fastq files. The reads are sampled from two custom haploid reference sequences with a unique set of heterozygous SNPs in order to facilitate the evaluation of allele-specific copy numbers. The haploid reference sequences were constructed as follows: first, we obtained a VCF file containing a large set of human genomic variants from the ncbi SNP database (accessable at `https://ftp.ncbi.nih.gov/snp/organisms/human\_9606/VCF/`). We filtered the VCF file for heterozygous SNPs using bcftools [3]. Second, we randomly selected 4.5 million from the remaining SNPs and distributed them over two sets A and B also at random as this figure is inline with estimates for the number of SNPs in the human genome [1]. Third, we constructed a haploid reference genome containing the variants in set A (resp. B) using the hg38 reference as a template using the GATK toolkit [15].

To prepare the raw fastq files for the methods, we applied standard preprocessing steps. Specifically, we first aligned them to the hg38 reference genome using BWA [9], then applied filtering and sorting with samtools [3] to generate the desired BAM files. Additionally, the BAF estimation procedure of CHISEL, which is also employed by SEACON, requires a file containing the position and phase of SNPs in the sampled individual. This was assembled by randomly sampling N SNPs from the original 4.5 million with phases set according to which reference genome it was inserted. We set N to be 1.6 million as this is the number that can be phased reliably using current reference-based phasing tools [2]. For context, we evaluated accuracy of the estimated mBAFs by measuring their RMSE with what is expected by the ground truth copy numbers. We found that 100 cells with 1Mbp bins had a mean RMSE of 0.1 at 0.05X coverage, increasing to 0.07 at 0.1X coverage, 0.06 for 0.25X coverage, and 0.04 for 0.5X coverage.

We generated 31 of these datasets which can broadly be categorized into two groups based on the number of cells; datasets A1-A16 all contain 100 cells, while datasets B1-B15 contain 500 or 1000 cells, with one dataset, B1, containing 5000 cells. The datasets explore various parameter values including bin sizes, ploidy and WGD, coverage, chromosomal rates, region length, and normal cell fraction. For coverage, we explore different cell-specific mean coverage but also the effects of coverage non-uniformity. Here, coverage non-uniformity is modelled with a beta distribution defined by a point (x, y) on the lorenz curve (see [18]). The region length essentially effects how close CNAs occur along the boundaries of the predefined bins, where the larger the regions, the easier it should be to detect discrete copy number segments (by default CNAs are simulated in units of 10,000 base pairs, but we explore increasing this to be comparable to the bin size). The normal cell fraction defines the percent of the total sampled cells which harbor no CNAs. See Tables S2 and S3 for the parameter values used for each dataset.

S4 Other methods

We included in our simulation study the five methods SCOPE [16], SeCNV [11], SCONCE [7], HMMCopy [13], Alleloscope [19], SIGNALS [62022Funnell et al.], and CHISEL [20]. All methods were run following the instructions provided in their respective manuals and use default parameters when possible. We briefly describe how each method was run as follows.

To run SCOPE, we ran the `get_bam_bed` function using hg38 and the resolution set to the bin size. We then ran the `get_mapp`, `get_gc`, `get_coverage_scDNA`, `get_samp_QC`, `perform_qc`, and `get_gini` functions exactly as in the tutorial. Next, we ran the `normalize_codex2_ns_noK` with gini coefficient threshold set to 0.12, and finished the normalization procedure by running `initialize_ploidy` and `normalize_scope_foreach` exactly as in the tutorial. For some datasets, we manually changed the gini coefficient threshold so that the number of identified normal cells was $\geq 50\%$ of the true number. Lastly, we ran the `segment_CBScs` function over each chromosome.

To run SeCNV, we obtained the BigWigOverBed file provided from the github repository and executed the `SeCNV.py` script over the bam files with the bin size and reference genome set appropriately.

To run SCONCE, we employed the list of diploid cells identified by SEACON and then ran `avgDiploid.R` and `fitMeanVarRlnshp.R` to generate average diploid cell depth, as well as the mean and variance relationship coefficients of diploid cells, in accordance with the guidelines provided in the documentation. Finally, we ran the `sconce` main function with k set to 10 for the final outcomes. It is important to note that this method does not converge for some cells. Therefore, only the results that predict over 50% of the cells successfully are considered.

To run HMMCopy, we used the `correctReadcount` for data preprocessing and then excuted `HMMsegment` using the default parameters to obtain the final results.

Before running the allele-specific profilers Alleloscope, SIGNALS, and CHISEL, we extracted the reference and alternate read counts at SNPs specified by the input vcf file according to the ground truth phase. The phased SNP count file was used to prepare the inputs for all three methods.

To run Alleloscope, we first prepared the five input files as specified by the instructions. We used the normalized readcounts and normal cells inferred by SEACON to create the tumor and normal read count matrix files. Next, the phased SNP count file was split into a reference count and alternate count file following the required format. With the inputs ready, we followed the tutorial titled *scDNA-seq with 2nd-stage estimation* on the github repository. We set the SNP filter to 1 and the min vaf and max vaf to 0 and 1, respectively.

To run SIGNALS, we initialized the two input dataframes manually. The input CN object was obtained by transforming the output files of HMMCopy, following the guidance of the author. The input haplotype object was initialized by dividing the genome into haplotype blocks of 250kbp, and grouping the reference and alternate counts in the phased SNP count file into those blocks. Then, the `callAlleleSpecificCN` command was used to generate the results.

To run CHISEL, we first ran the `chisel_combocall` program on the RDRs estimated by SEACON with the phased SNP count file to obtain the BAF estimates. For all experiments, we used a haplotype block size of $250kbp$. Then, the `chisel_calling` program was used on the RDRs and BAFs to generate the copy number profiles.

Table S1. Parameters used in the direct read count and BAF simulations.

Simulation	Scale factor	Read count noise rate	BAF noise rate
C1	100	0	0.01
C2	300	0	0.01
C3	600	0	0.01
C4	100	0.1	0.03
C5	300	0.1	0.03
C6	600	0.1	0.03

Table S2. Parameters used to generate the synthetic DNA sequencing read simulations in group A, which all have 100 cells. The table lists the main simulation parameters in coverage, bin size, and ploidy, as well as any additional information that makes the dataset unique. Additional information on the simulation model and parameters appears in Supplemental Section S3, but a full description is given in [18].

dataset id	# cells	coverage	bin size	ploidy	notes
A1	100	0.1X	1Mbp	~2	First representative dataset
A2	100	0.1X	1Mbp	~4	Second representative dataset
A3	100	0.05X	1Mbp	~2	
A4	100	0.25X	1Mbp	~2	
A5	100	0.5X	1Mbp	~2	Very high coverage
A6	100	0.25X	1Mbp	~4	
A7	100	0.1X	5Mbp	~2	
A8	100	0.1X	5Mbp	~2	Region length of $1Mbp$
A9	100	0.1X	5Mbp	~2	Region length of $5Mbp$
A10	100	0.02X	5Mbp	~2	
A11	100	0.05X	5Mbp	~2	
A12	100	0.1X	1Mbp	~2	Normal cell fraction 10%
A13	100	0.1X	1Mbp	~2	Normal cell fraction 40%
A14	100	0.1X	1Mbp	~2	Nonuniform coverage $(x = 0.5, y = 0.38)$
A15	100	0.1X	1Mbp	~2	Nonuniform coverage $(x = 0.5, y = 0.27)$
A16	100	0.1X	0.5Mbp	~2	Small bins

S5 Supplementary Tables and Figures

Table S3. Parameters used to generate the synthetic DNA sequencing read simulations in group B, which have more than 100 cells. The table lists the main simulation parameters in number of cells, coverage, bin size, and ploidy, as well as any additional information that makes the dataset unique. Additional information on the simulation model and parameters appears in Supplemental Section S3, but a full description is given in [18].

dataset id	# cells	coverage	bin size	ploidy	notes
B1	5000	0.02X	5Mbp	~2	5k cells, third representative dataset
B2	1000	0.02X	5Mbp	~2	
B3	1000	0.05X	5Mbp	~2	
B4	1000	0.1X	1Mbp	~2	
B5	1000	0.02X	5Mbp	~3.5	
B6	1000	0.05X	5Mbp	~3.5	
B7	500	0.02X	5Mbp	~2	
B8	500	0.05X	5Mbp	~1.5	
B9	500	0.1X	1Mbp	~2	
B10	500	0.02X	5Mbp	~4	
B11	500	0.05X	5Mbp	~4	
B12	1000	0.05X	5Mbp	~4	balanced chromosomal gains/losses
B13	1000	0.05X	5Mbp	~4.5	more chromosomal gain, less deletion
B14	1000	0.02X	5Mbp	~2	mean 5 CNAs per edge
B15	1000	0.02X	5Mbp	~2	mean 8 CNAs per edge

Table S4. Breakpoint performance of GMM when breakpoints are filtered to those present in $\geq t$ cells.

Dataset	Noise	Scale	$\mathbf{t = 1}$		$\mathbf{t = 5}$		$\mathbf{t = 10}$	
			Precision	Recall	Precision	Recall	Precision	Recall
D1	Low	100	0.911	0.963	0.999	0.778	0.999	0.678
D2	Low	300	0.972	0.965	0.998	0.775	1.0	0.684
D3	Low	600	0.984	0.967	1.0	0.748	1.0	0.655
D4	High	100	0.547	0.877	0.886	0.701	0.971	0.615
D5	High	300	0.632	0.876	0.917	0.704	0.981	0.614
D6	High	600	0.645	0.879	0.926	0.704	0.986	0.617

Table S5. Breakpoint performance of each component of SEACON on directly simulated RDR and BAFs in comparison to Kmeans clustering used by CHISEL and CBS alone. Note that the GMM alone is equivalent to using SEACON with $t = 1$.

Dataset	Noise	Scale	k-means		CBS Only		GMM Only		SEACON $(t = 5)$	
			Precision	Recall	Precision	Recall	Precision	Recall	Precision	Recall
D1	Low	100	0.886	0.965	0.853	0.829	0.911	0.963	0.984	0.929
D2	Low	300	0.905	0.962	0.864	0.837	0.972	0.965	0.985	0.930
D3	Low	600	0.949	0.965	0.860	0.840	0.984	0.967	0.985	0.930
D4	High	100	0.561	0.882	0.790	0.757	0.547	0.877	0.880	0.834
D5	High	300	0.581	0.886	0.800	0.766	0.632	0.876	0.902	0.842
D6	High	600	0.592	0.887	0.794	0.772	0.645	0.879	0.911	0.840

Table S6. Precision and recall values for breakpoint detection of Simulation A1 over increasing tolerance (distance in bins between the predicted and true breakpoint for it to be counted as correct). Reported values are the averages over the individual precision/recall of each cell. SEACON1 represents setting $t = 1$ and SEACON5 represents setting $t = 5$.

	Tolerance	SCOPE	SeCNV	SCONCE	HMMCopy	Alleloscope	SIGNALS	CHISEL	SEACON1	SEACON5
Precision	0	0.74	0.866	0.746	0.776	-	0.205	0.579	0.651	0.761
	1	0.912	0.959	0.97	0.959	-	0.284	0.615	0.863	0.981
	2	0.973	0.966	0.87	0.959	-	0.299	0.624	0.867	0.984
Recall	0	0.353	0.362	0.709	0.282	-	0.375	0.3	0.635	0.604
	1	0.433	0.399	0.925	0.346	-	0.524	0.334	0.867	0.849
	2	0.46	0.402	0.926	0.346	-	0.553	0.358	0.870	0.852

Table S7. Precision and recall values for breakpoint detection of Simulation A2 over increasing tolerance (distance in bins between the predicted and true breakpoint for it to be counted as correct). Reported values are the averages over the individual precision/recall of each cell. SEACON1 represents setting $t = 1$ and SEACON5 represents setting $t = 5$.

	Tolerance	SCOPE	SeCNV	SCONCE	HMMCopy	Alleloscope	SIGNALS	CHISEL	SEACON1	SEACON5
Precision	0	0.616	0.763	0.53	0.691	-	0.547	0.429	0.522	0.601
	1	0.924	0.932	0.691	0.972	-	0.716	0.595	0.806	0.977
	2	0.971	0.969	0.705	0.974	-	0.742	0.608	0.809	0.977
Recall	0	0.312	0.094	0.2	0.141	-	0.167	0.27	0.499	0.439
	1	0.67	0.113	0.263	0.201	-	0.22	0.382	0.834	0.753
	2	0.491	0.118	0.268	0.201	-	0.229	0.391	0.839	0.753

Table S8. Precision and recall values for breakpoint detection of Simulation B1 over increasing tolerance (distance in bins between the predicted and true breakpoint for it to be counted as correct). Reported values are the averages over the individual precision/recall of each cell. SEACON1 represents setting $t = 1$ and SEACON5 represents setting $t = 5$.

	Tolerance	SCOPE	SeCNV	SCONCE	HMMCopy	Alleloscope	SIGNALS	CHISEL	SEACON1	SEACON5
Precision	0	0.381	0.558	0.57	0.882	-	0.393	0.394	0.538	0.463
	1	0.531	0.854	0.803	0.966	-	0.748	0.636	0.726	0.669
	2	0.544	0.906	0.818	0.99	-	0.777	0.659	0.739	0.686
Recall	0	0.237	0.237	0.432	0.086	-	0.15	0.197	0.508	0.438
	1	0.363	0.363	0.614	0.093	-	0.286	0.315	0.686	0.63
	2	0.385	0.385	0.625	0.095	-	0.297	0.328	0.698	0.646

Table S9. Performance of using CHISEL with the breakpoint filtering heuristic compared to standard CHISEL over datasets A1, A2, and B1. The heuristic involves first replacing the GMM results in SEACON with the inferred bin clusters from CHISEL, then running the remainder of the SEACON pipeline to infer final segments and copy numbers. Reported values are the average of the mean F1-score of each cell for a distance tolerance of l bins between the predicted and true breakpoints.

	A1			A2			B1		
	$l = 0$	$l = 1$	$l = 2$	$l = 0$	$l = 1$	$l = 2$	$l = 0$	$l = 1$	$l = 2$
CHISEL	0.351	0.382	0.39	0.314	0.442	0.452	0.247	0.397	0.412
CHISEL w/ Heuristic	0.395	0.407	0.409	0.331	0.455	0.465	0.233	0.393	0.423

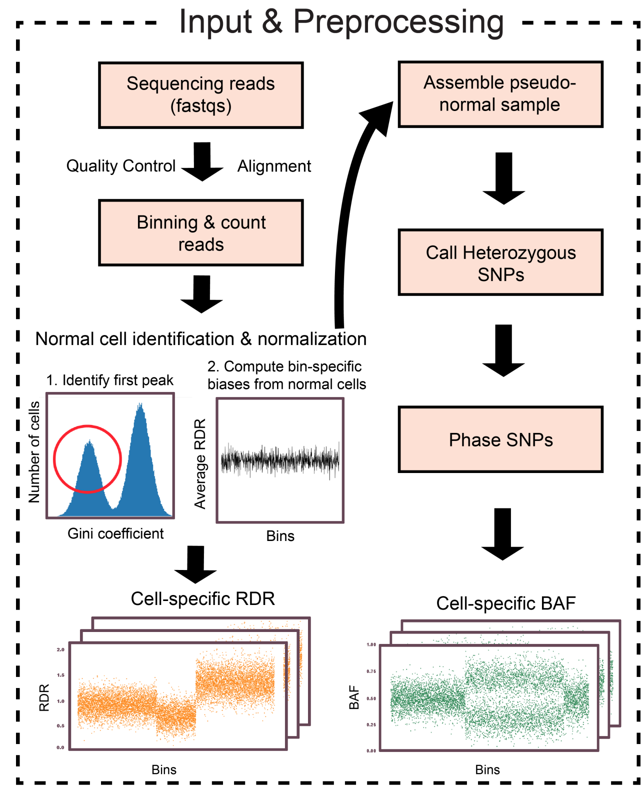

Fig. S1. The preprocessing workflow of SEACON. After standard aligning and filtering raw reads, SEACON identifies normal cells using the gini coefficient and uses them as negative controls for computing corrected read-depth ratios (RDRs). Reads from the normal cells are combined to form a pseudo-normal sample used for identify heterozygous SNPs and computing B-allele frequencies (BAFs).

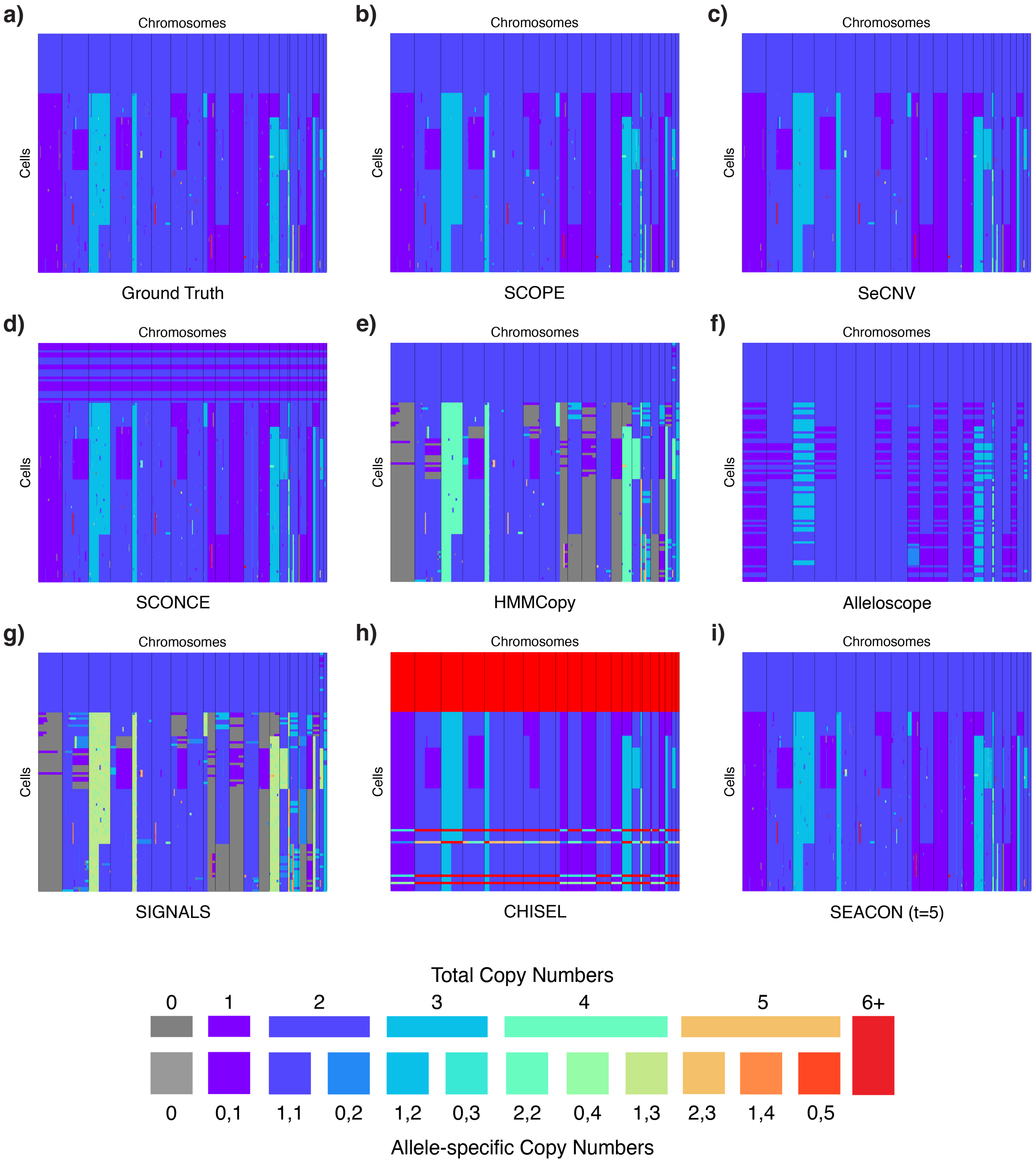

Fig. S2. A visualization of dataset A1 (100 cells, 0.1X coverage, 1Mbp bins) through heatmaps of the genome-wide ground truth copy number profiles and those returned by the methods. Results are shown across a common set of 2452 bins out of a possible 2887 and all 100 cells. The heatmaps of the ground truth (**a**), Alleloscope (**f**), SIGNALS (**g**), CHISEL (**h**), and SEACON (**i**) all show allele-specific copy numbers, whereas the heatmaps of SCOPE (**b**), SeCNV (**c**), SCONCE (**d**), and HMMCopy (**e**) show total copy numbers.

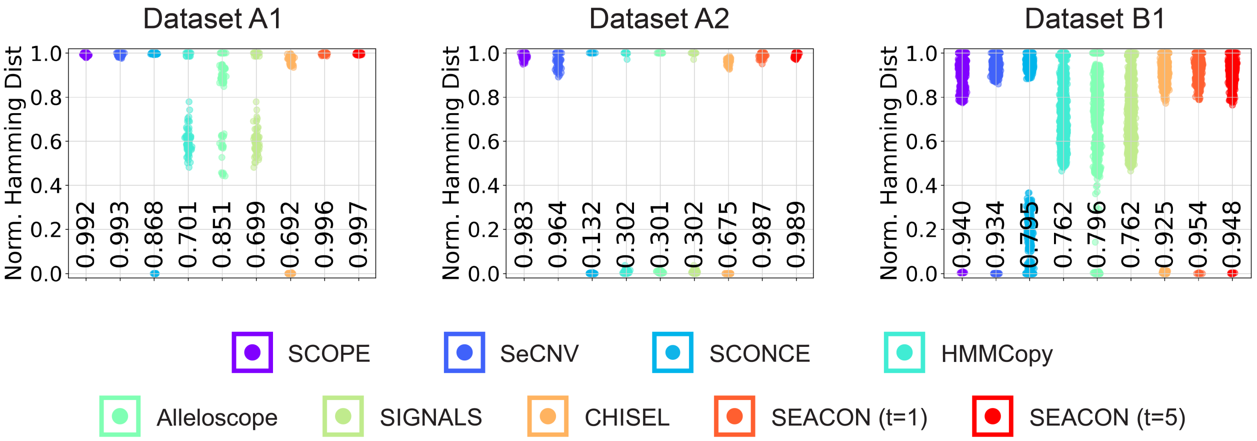

Fig. S3. The fraction of the genome with correct copy number inferences, equivalent to the normalized hamming distance, of SEACON and the 7 other methods on the three representative datasets A1, A2, and B3. Each dot represents the mean value across all bins of a cell, and the mean across all bins from every cell is printed for each method.

References

1. D. R. Bentley. The human genome project—an overview. *Medicinal research reviews*, 20(3):189–196, 2000.
2. Y. Choi, A. P. Chan, E. Kirkness, A. Telenti, and N. J. Schork. Comparison of phasing strategies for whole human genomes. *PLoS genetics*, 14(4):e1007308, 2018.
3. P. Danecek, J. K. Bonfield, J. Liddle, J. Marshall, V. Ohan, M. O. Pollard, A. Whitwham, T. Keane, S. A. McCarthy, R. M. Davies, and H. Li. Twelve years of SAMtools and BCFtools. *Gigascience*, 10(2), Feb. 2021.
4. R. Desper and O. Gascuel. Fast and accurate phylogeny reconstruction algorithms based on the minimum-evolution principle. In R. Guigó and D. Gusfield, editors, *Algorithms in Bioinformatics*, pages 357–374, Berlin, Heidelberg, 2002. Springer Berlin Heidelberg.
5. X. Dong, L. Zhang, X. Hao, T. Wang, and J. Vijg. Sccnv: a software tool for identifying copy number variation from single-cell whole-genome sequencing. *Frontiers in Genetics*, 11:505441, 2020.
6. T. Garvin, R. Aboukhalil, J. Kendall, T. Baslan, G. S. Atwal, J. Hicks, M. Wigler, and M. C. Schatz. Interactive analysis and assessment of single-cell copy-number variations. *Nature methods*, 12(11):1058–1060, 2015.
7. S. Hui and R. Nielsen. SCONCE: a method for profiling copy number alterations in cancer evolution using single-cell whole genome sequencing. *Bioinformatics*, 38(7):1801–1808, 01 2022.
8. T. L. Kaufmann, M. Petkovic, T. B. Watkins, E. C. Colliver, S. Laskina, N. Thapa, D. C. Minussi, N. Navin, C. Swanton, P. Van Loo, et al. Medicc2: whole-genome doubling aware copy-number phylogenies for cancer evolution. *Genome biology*, 23(1):241, 2022.
9. H. Li and R. Durbin. Fast and accurate long-read alignment with burrows–wheeler transform. *Bioinformatics*, 26(5):589–595, 2010.
10. A. B. Olshen, E. S. Venkatraman, R. Lucito, and M. Wigler. Circular binary segmentation for the analysis of array-based dna copy number data. *Biostatistics*, 5(4):557–572, 2004.
11. W. Ruohan, Z. Yuwei, W. Mengbo, F. Xikang, W. Jianping, and L. Shuai Cheng. Resolving single-cell copy number profiling for large datasets. *Briefings in Bioinformatics*, 23(4):bbac264, 2022.
12. R. F. Schwarz, A. Trinh, B. Sipos, J. D. Brenton, N. Goldman, and F. Markowetz. Phylogenetic quantification of intra-tumour heterogeneity. *PLoS computational biology*, 10(4):e1003535, 2014.
13. S. P. Shah, X. Xuan, R. J. DeLeeuw, M. Khojasteh, W. L. Lam, R. Ng, and K. P. Murphy. Integrating copy number polymorphisms into array CGH analysis using a robust HMM. *Bioinformatics*, 22(14):e431–e439, 07 2006.
14. Z. Sondka, S. Bamford, C. G. Cole, S. A. Ward, I. Dunham, and S. A. Forbes. The cosmic cancer gene census: describing genetic dysfunction across all human cancers. *Nature Reviews Cancer*, 18(11):696–705, 2018.
15. G. Van der Auwera and O. BD. *Genomics in the Cloud: Using Docker, GATK, and WDL in Terra (1st Edition)*. O'Reilly Media, 2020.
16. R. Wang, D.-Y. Lin, and Y. Jiang. Scope: a normalization and copy-number estimation method for single-cell dna sequencing. *Cell systems*, 10(5):445–452, 2020.

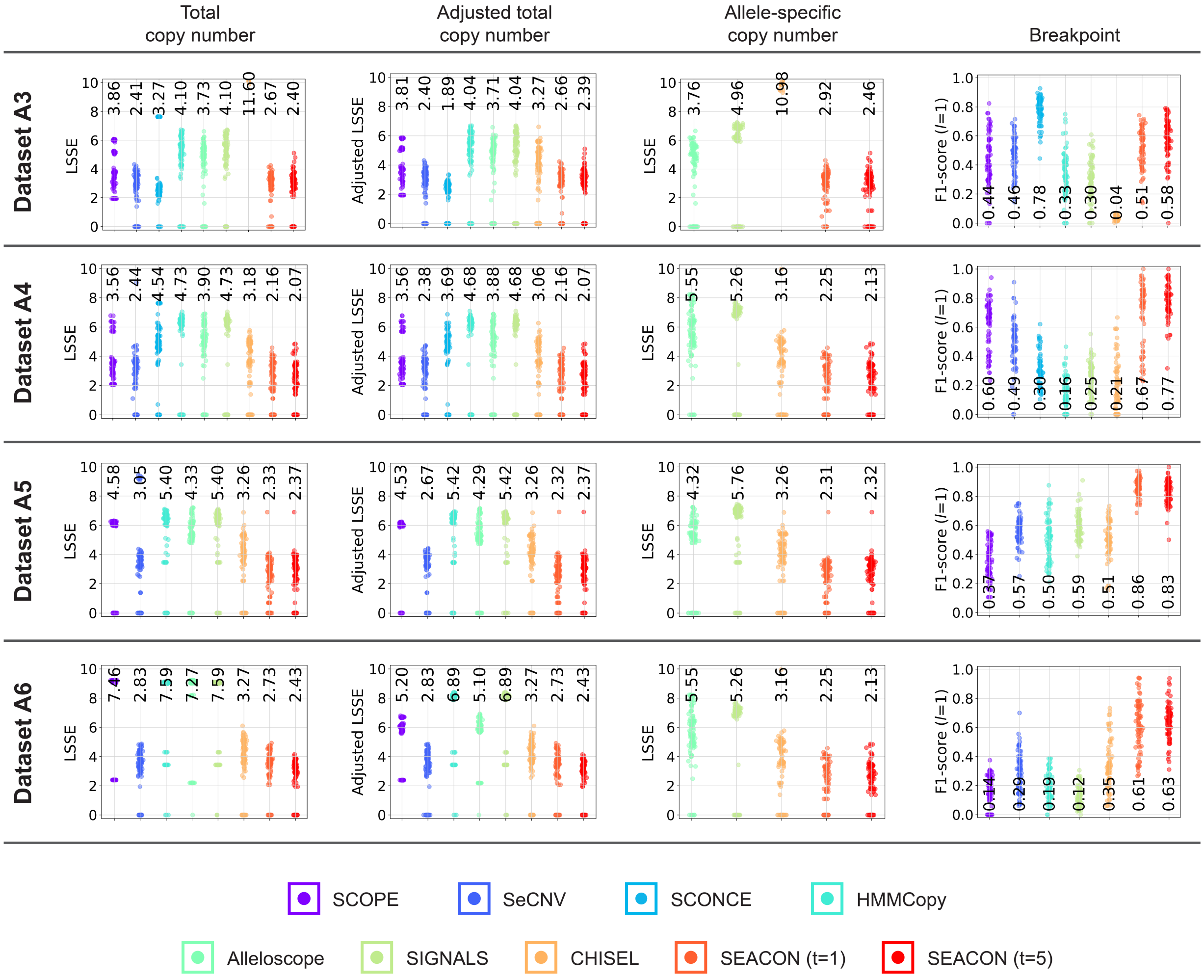

Fig. S4. Results of SEACON and the seven existing CNA detection algorithms over simulated datasets A3-A6: A3 (first row, 100 cells, 0.05X coverage), A4 (second row, 100 cells, 0.25X coverage), A5 (third row, 100 cells, 0.5X coverage), and A6 (fourth row, 100 cells, 0.25X coverage, tetraploid). See Table S2 for further details. Results from a method are omitted if they failed to produce outputs on the dataset or if the results covered $\leq 50\%$ of the total cells and bins.

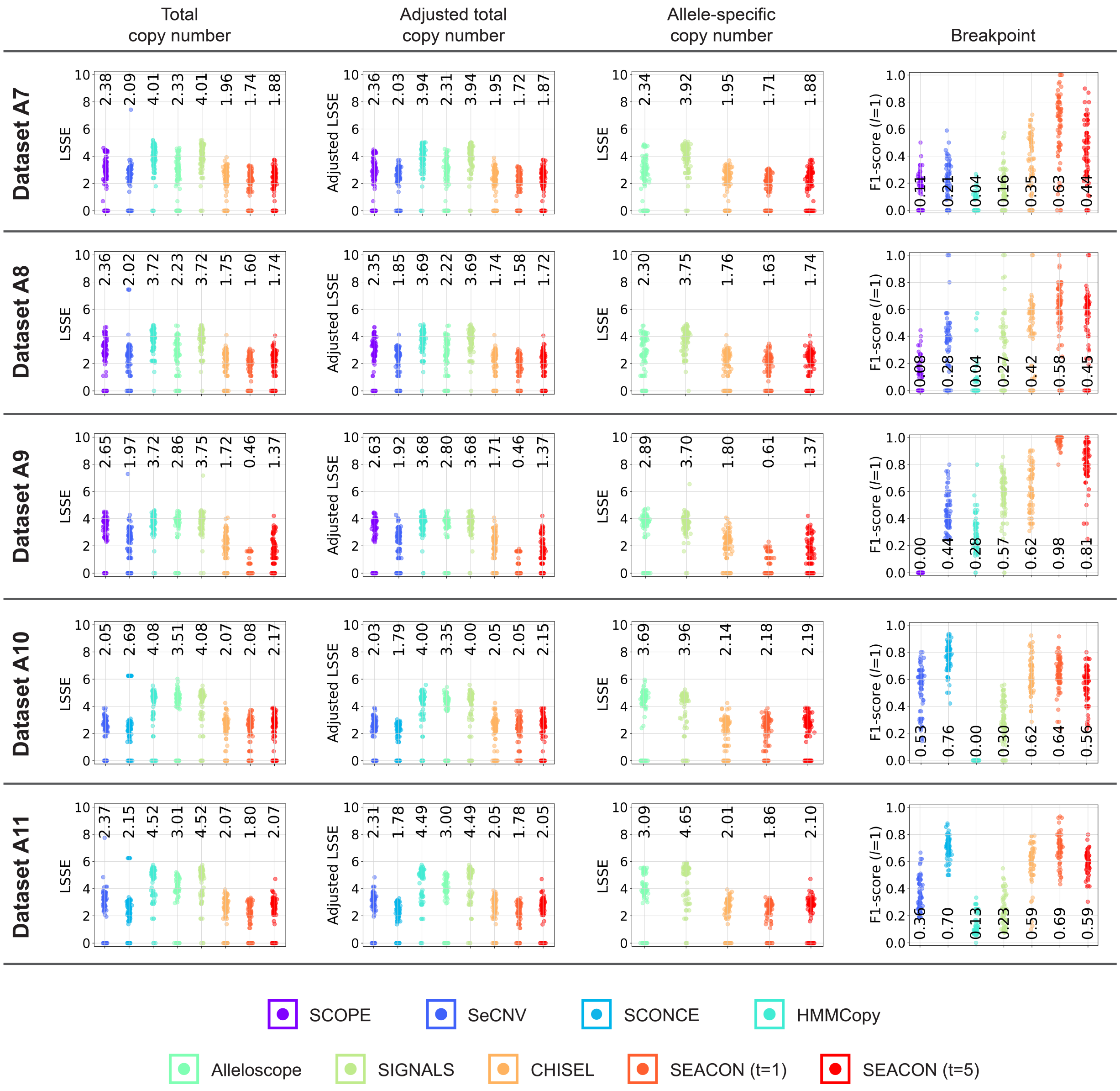

Fig. S5. Results of SEACON and the seven existing CNA detection algorithms over simulated datasets A7-A11: A7 (first row, 100 cells, 0.1X coverage), A8 (second row, 100 cells, 0.1X coverage, region length 1Mbp), A9 (third row, 100 cells, 0.1X coverage, region length 5Mbp), A10 (fourth row, 100 cells, 0.02X coverage), and A11 (fifth row, 100 cells, 0.05X coverage). See Table S2 for further details. Results from a method are omitted if they failed to produce outputs on the dataset or if the results covered $\leq 50\%$ of the total cells and bins.

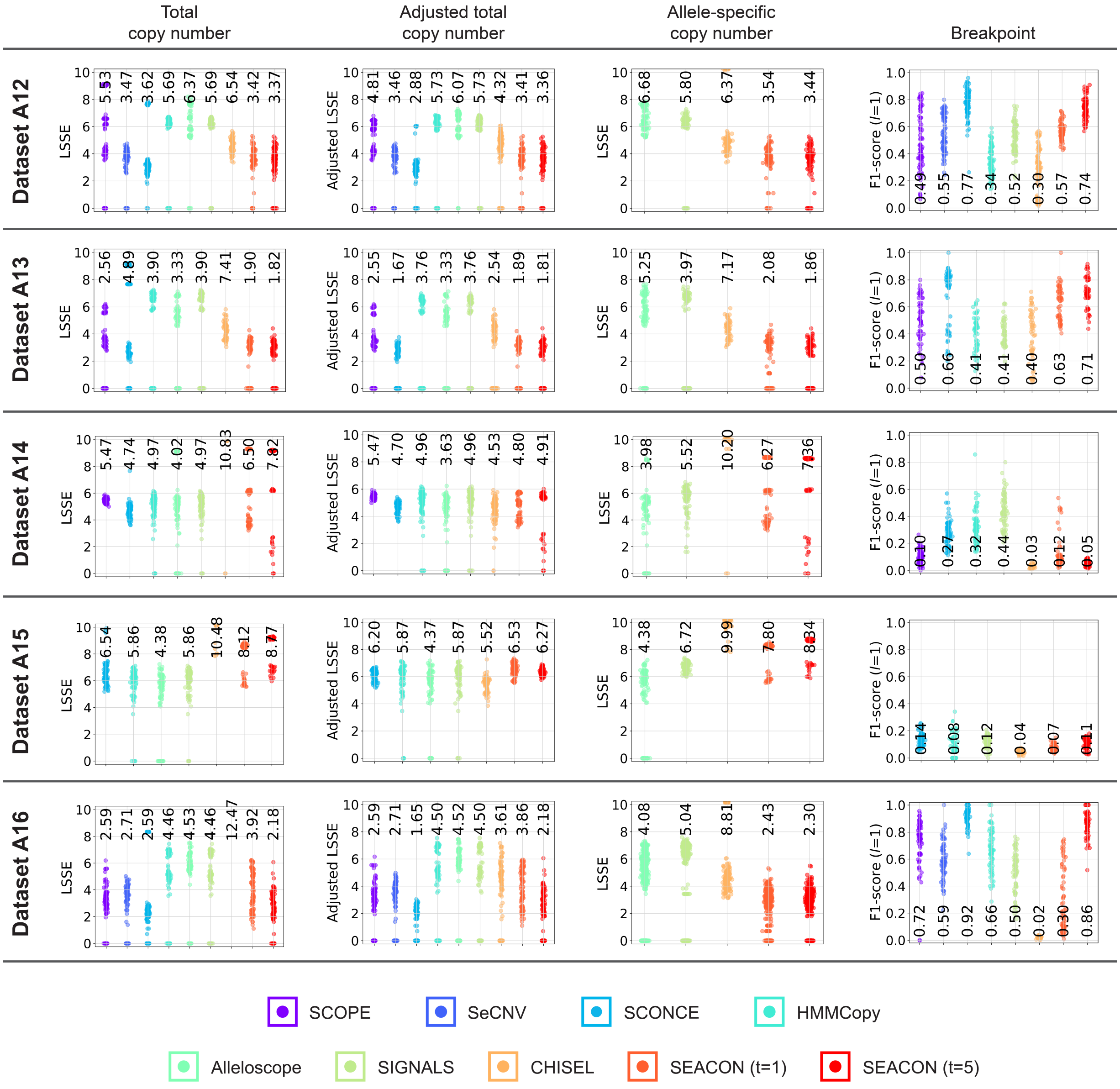

Fig. S6. Results of SEACON and the seven existing CNA detection algorithms over simulated datasets A12-A16: A12 (first row, 100 cells, 0.1X coverage, 10% normal cells), A13 (second row, 100 cells, 0.1X coverage, 40% normal cells), A14 (third row, 100 cells, 0.1X coverage, extreme coverage non-uniformity), A15 (fourth row, 100 cells, 0.1X coverage, extreme coverage non-uniformity), and A16 (fifth row, 100 cells, 0.1X coverage, 500kbp bins). See Table S2 for further details. Results from a method are omitted if they failed to produce outputs on the dataset or if the results covered $\leq 50\%$ of the total cells and bins.

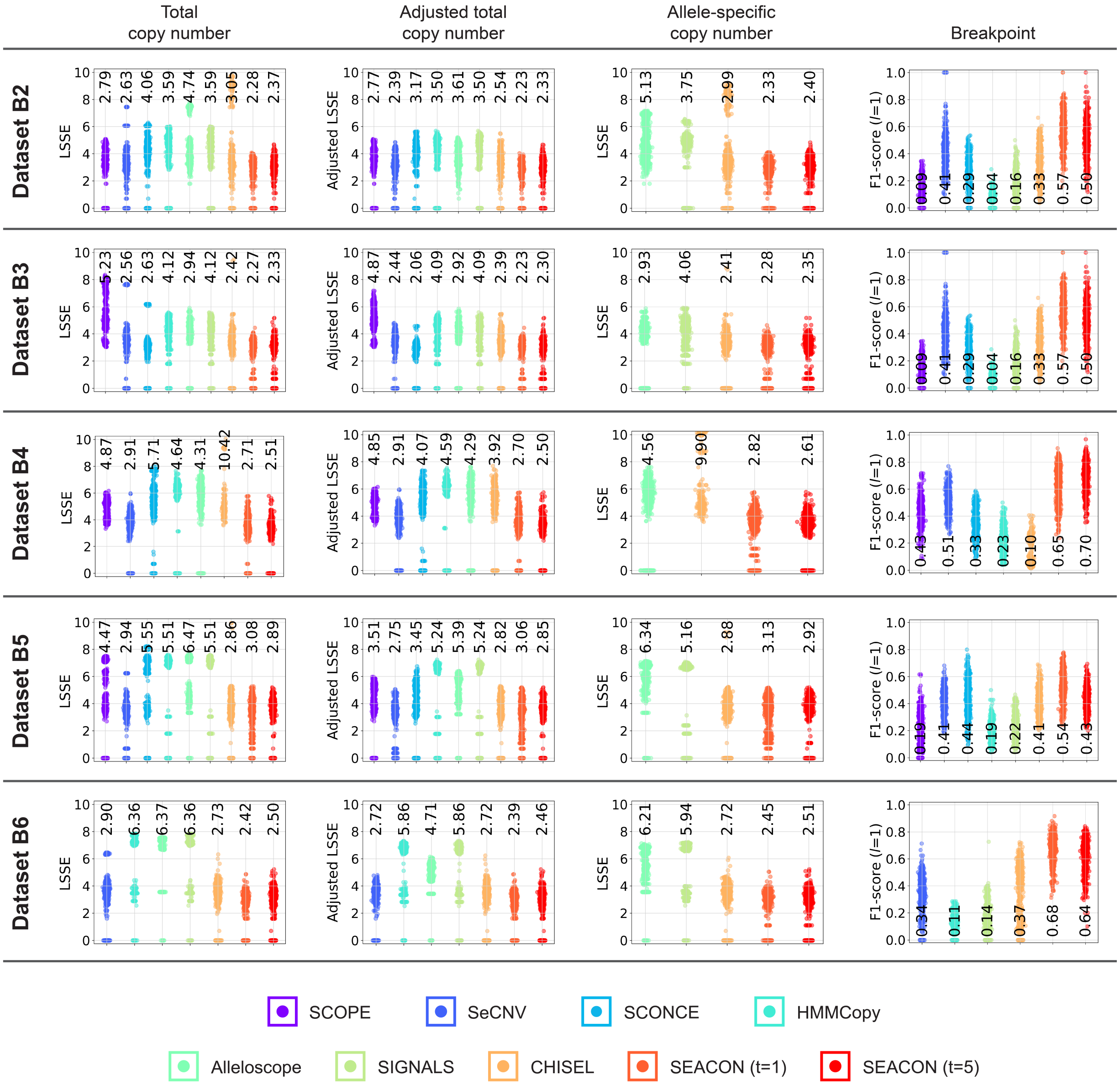

Fig. S7. Results of SEACON and the seven existing CNA detection algorithms over simulated datasets B2-B6: B2 (first row, 1k cells, 0.02X coverage), B3 (second row, 1k cells, 0.05X coverage), B4 (third row, 1k cells, 0.1X coverage), B5 (fourth row, 1k cells, 0.02X coverage, ploidy ~3.5), and B6 (fifth row, 1k cells, 0.05X coverage, ploidy ~3.5). See Table S3 for further details. Results from a method are omitted if they failed to produce outputs on the dataset or if the results covered $\leq 50\%$ of the total cells and bins.

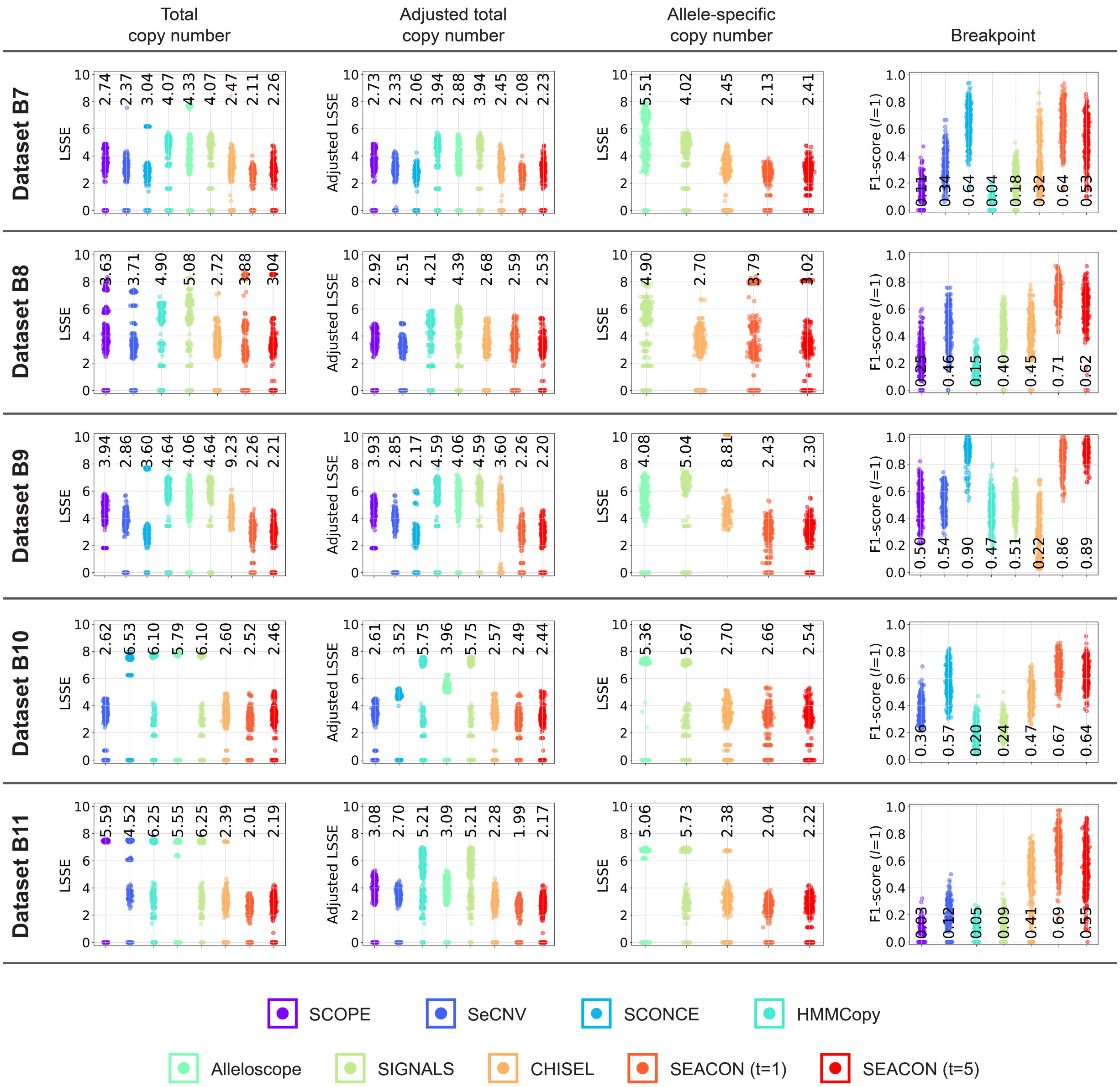

Fig. S8. Results of SEACON and the seven existing CNA detection algorithms over simulated datasets B7-B11: B7 (first row, 500 cells, 0.02X coverage), B8 (second row, 500 cells, 0.05X coverage, ploidy ~1.5), B9 (third row, 500 cells, 0.1X coverage), B10 (fourth row, 500 cells, 0.02X coverage, ploidy ~4), and B11 (fifth row, 500 cells, 0.05X coverage, ploidy ~4). See Table S3 for further details. Results from a method are omitted if they failed to produce outputs on the dataset or if the results covered $\leq 50\%$ of the total cells and bins.

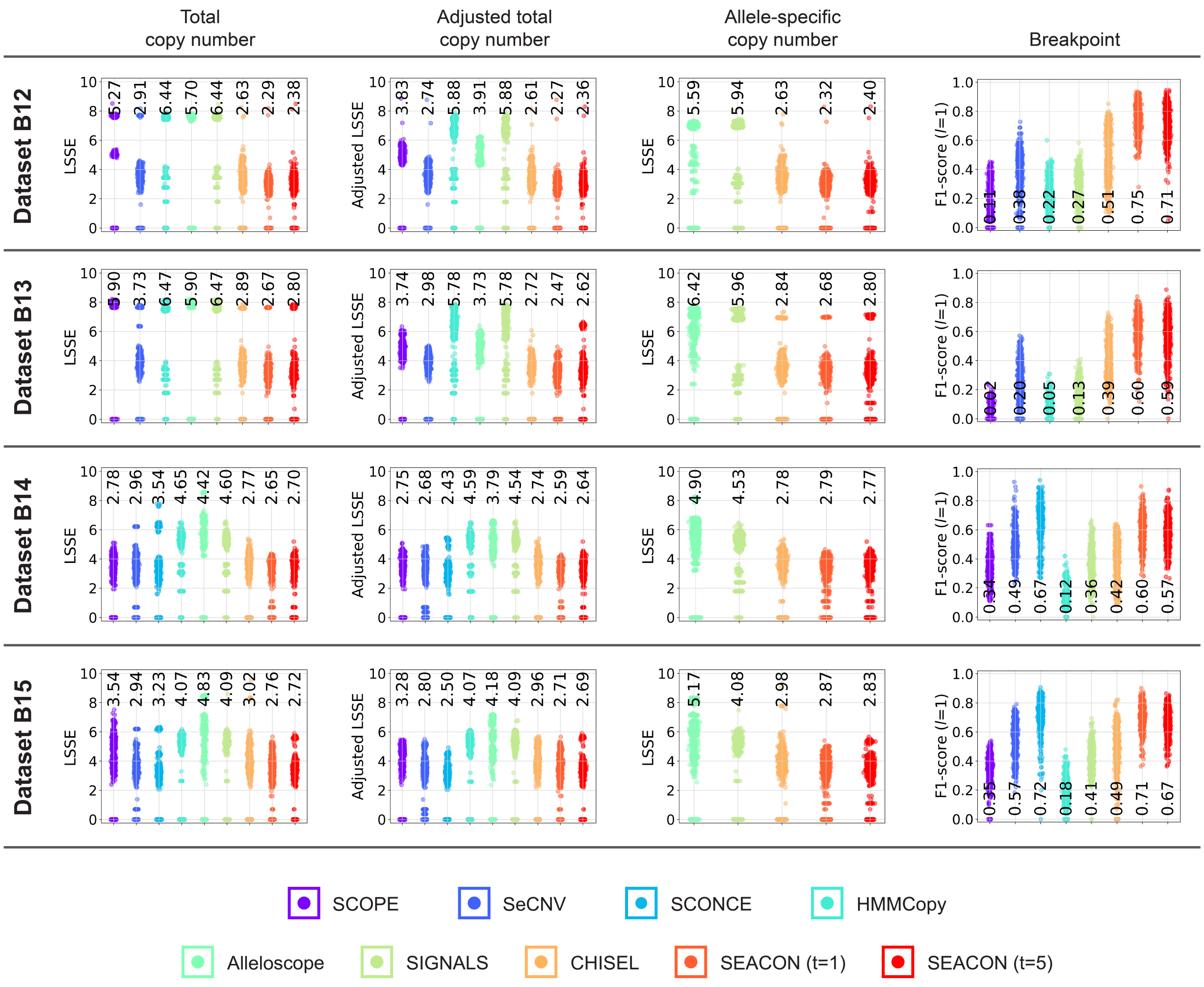

Fig. S9. Results of SEACON and the seven existing CNA detection algorithms over simulated datasets B12-B15: B12 (first row, 1k cells, 0.05X coverage), B13 (second row, 1k cells, 0.05X coverage, ploidy ~4.5), B14 (third row, 1k cells, 0.02X coverage, high CN rate), and B15 (fifth row, 1k cells, 0.02X coverage, high CN rate). See Table S3 for further details. Results from a method are omitted if they failed to produce outputs on the dataset or if the results covered $\leq 50\%$ of the total cells and bins.

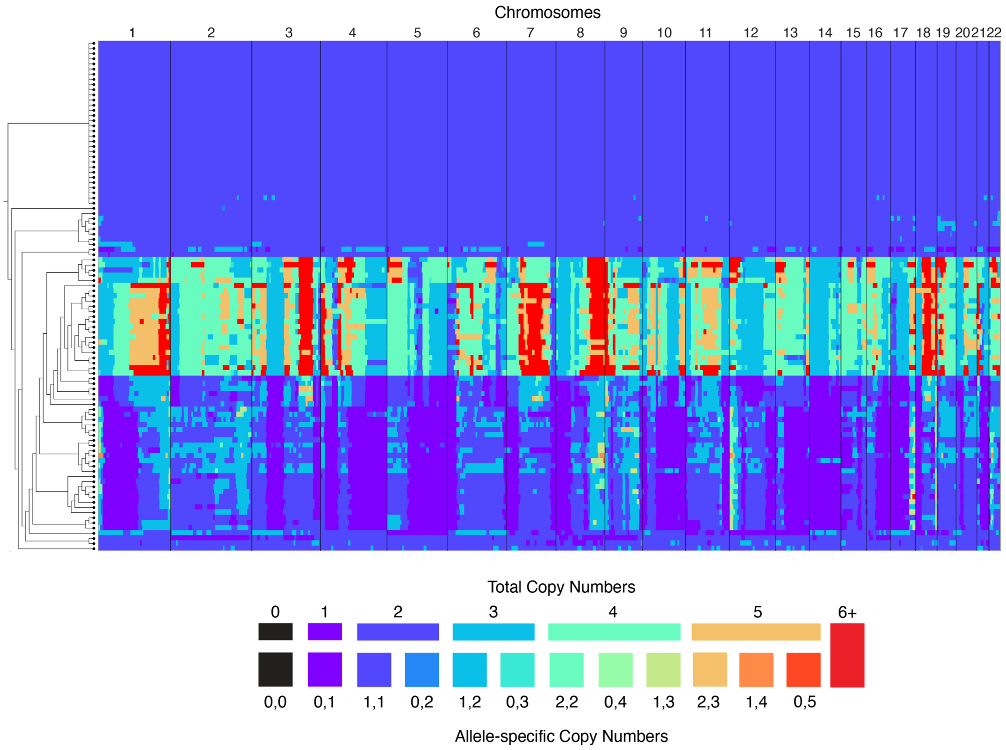

Fig. S10. Whole-genome allele-specific copy number profiles of patient T10 inferred by SEACON. A cell-lineage tree was constructed from pairwise distances computed over the allele-specific copy numbers under a model of CNA evolution.

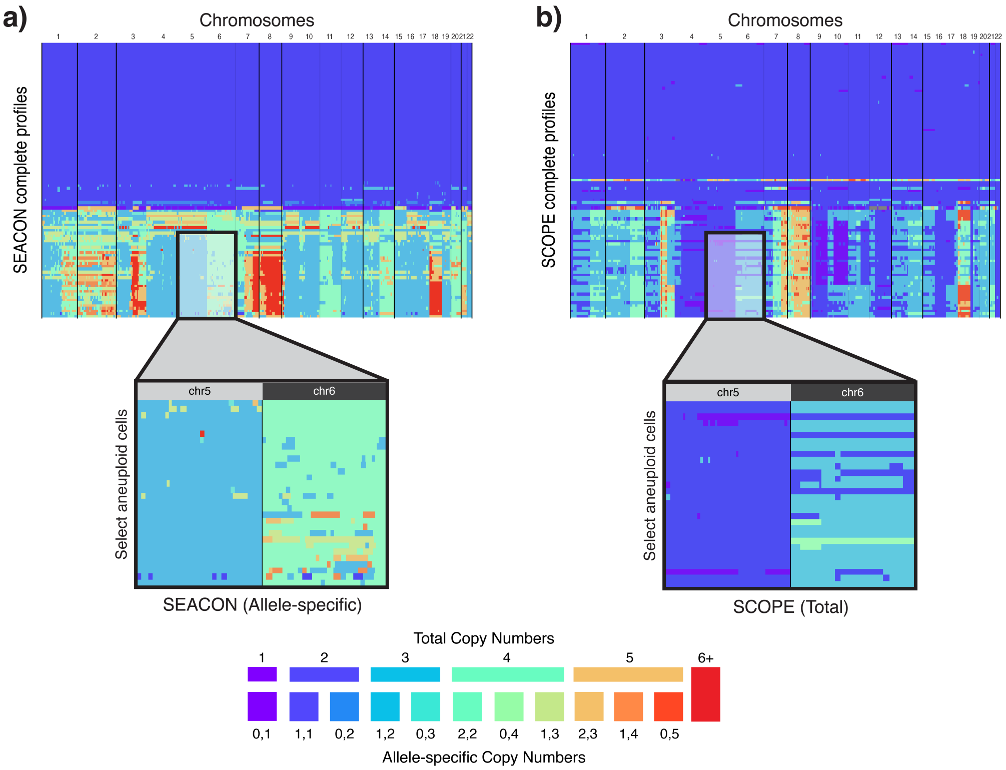

Fig. S11. Comparing regions of allelic balance vs imbalance in patient T16. **(a)** Heatmap of the allele-specific copy number profiles from SEACON for 38 aneuploid cells over chromosomes 5 and 6. The consensus total copy numbers of chromosomes 5 and 6 are 3 and 4, respectively, which suggests a state of allelic imbalance for chromosome 5 and balance for chromosome 6. **(b)** Heatmap of the total copy number profiles from SCOPE for the same cells and chromosomes. The consensus total copy numbers of chromosomes 5 and 6 are 2 and 3, respectively, which suggests the opposite states of allelic balance compared to SEACON.

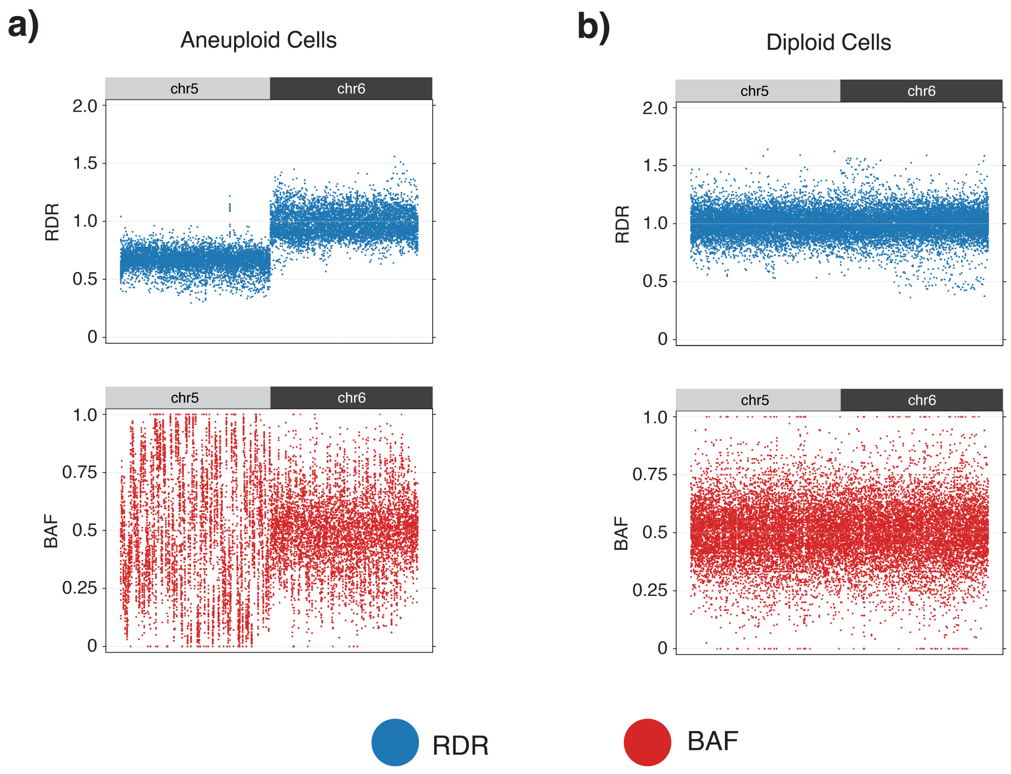

Fig. S12. Comparing regions of allelic balance vs imbalance in patient T16. **(c)** The RDR and BAF of bins for 38 aneuploid cells over chromosome 5 and 6, corresponding to the same cells and regions which illustrate the disagreement in output between SEACON and SCOPE (see Supplementary Figure S11). **(d)** The RDR and BAF of the normal diploid cells over the same region. Overall, the plots support the state of allelic balance implied by SEACON despite the BAF measurements being extremely noisy.

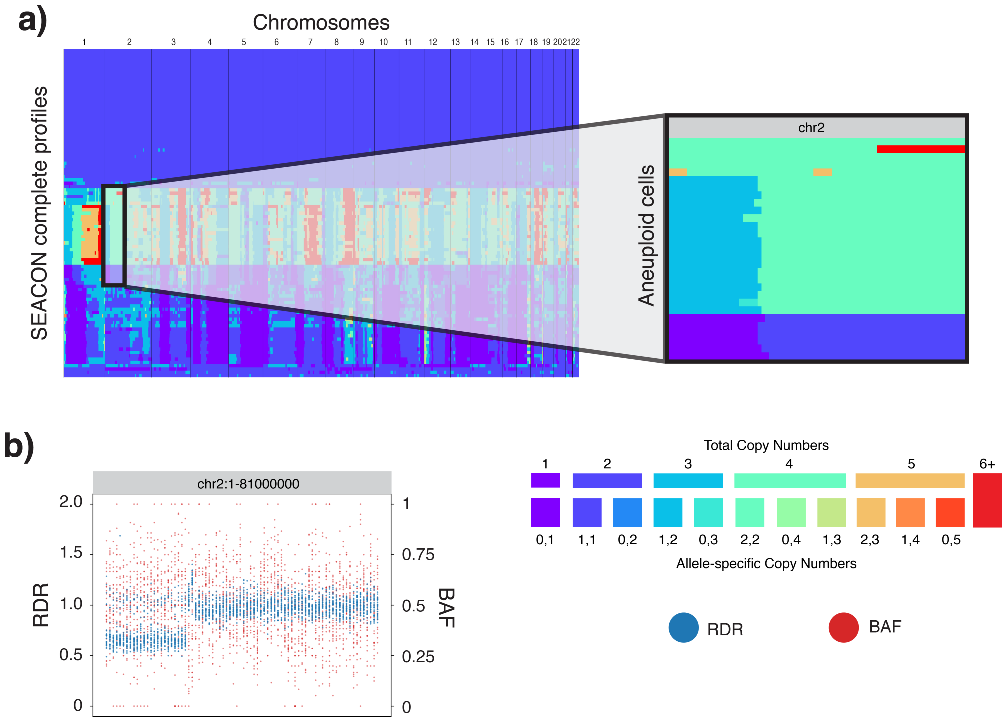

Fig. S13. Comparing regions of allelic balance vs imbalance in patient T10. **(a)** Heatmap of the 29 aneuploid cells over the first 80 bins of chromosome 2 (base pairs 1-81,000,000). The for the majority of these cells, the first 25 bins represent an unbalanced state ($\{0, 1\}$ or $\{1, 2\}$) while the remaining bins represent a balanced state ($\{1, 1\}$ or $\{2, 2\}$) By comparison, SCOPE returned a total copy number of 2 for bins 1-25 and 3 for bins 25-80. **(b)** RDRs and BAFs for the aneuploid cell along the same region. Unlike with patient T16, there is no discernible signal of allelic imbalance between bins 1-25 and 25-80. SEACON then assigns an unbalanced state and balanced state leading to roughly 1 less or 1 more than the true ploidy.

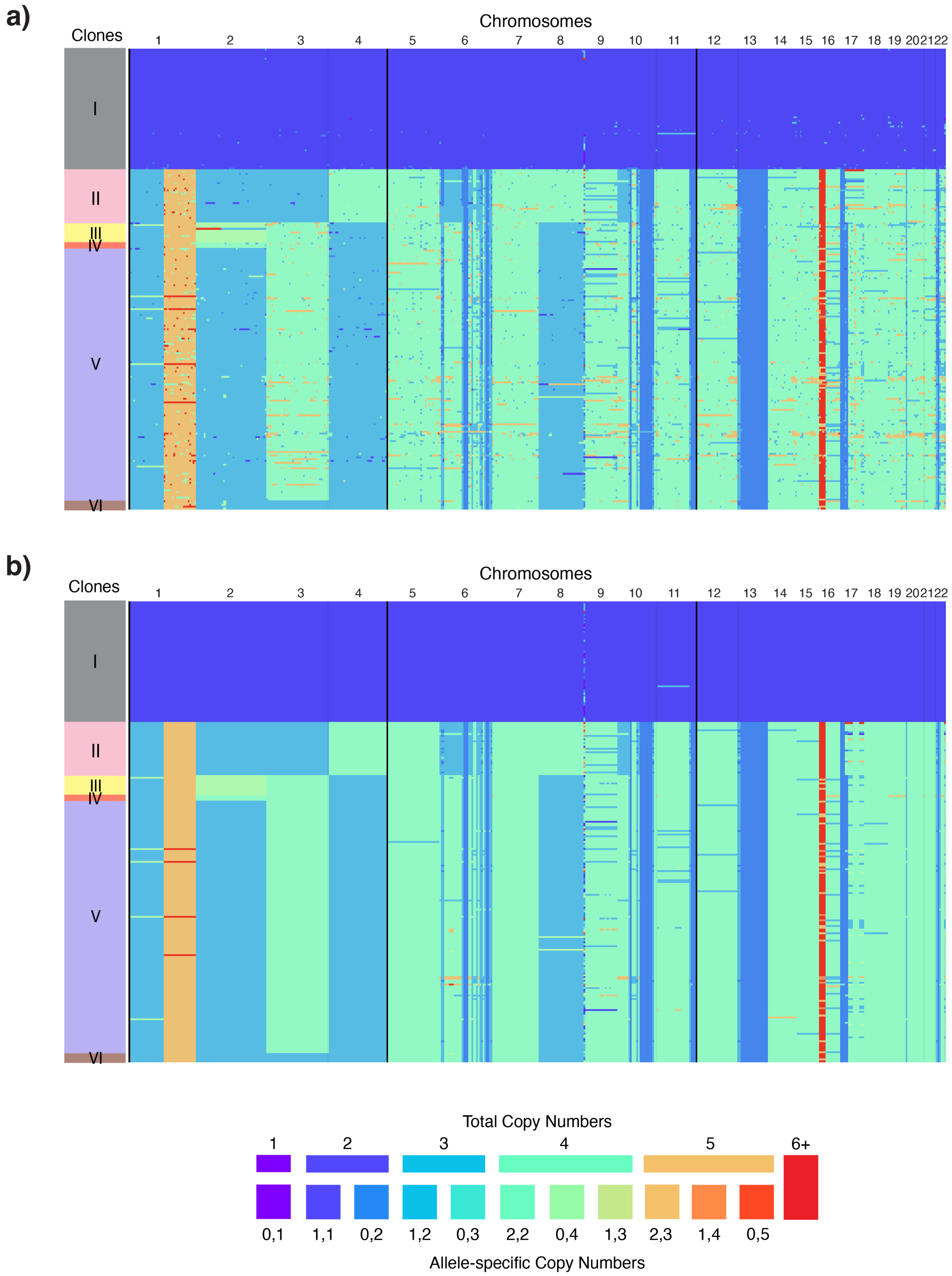

Fig. S14. Whole-genome allele-specific copy number profiles of a 10x genomics single-cell dataset. (**a**) Output profiles from SEACON. (**b**) Output profiles from CHISEL.

17. X. Wang, H. Chen, and N. R. Zhang. Dna copy number profiling using single-cell sequencing. *Briefings in bioinformatics*, 19(5):731–736, 2018.
18. S. Weiner and M. S. Bansal. Cnasim: improved simulation of single-cell copy number profiles and dna-seq data from tumors. *Bioinformatics*, 39(7):btad434, 2023.
19. C.-Y. Wu, B. T. Lau, H. S. Kim, A. Sathe, S. M. Grimes, H. P. Ji, and N. R. Zhang. Integrative single-cell analysis of allele-specific copy number alterations and chromatin accessibility in cancer. *Nature Biotechnology*, 39(10):1259–1269, Oct 2021.
20. S. Zaccaria and B. J. Raphael. Characterizing allele- and haplotype-specific copy numbers in single cells with chisel. *Nature Biotechnology*, 39(2):207–214, Feb 2021.
